# Capabilities, opportunities and motivations in implementing guideline-oriented biopsychosocial low back pain management: perceptions of occupational healthcare professionals after an educational intervention

**DOI:** 10.1186/s12913-025-13267-7

**Published:** 2025-08-29

**Authors:** Maija Tuulia Paukkunen, Riikka Holopainen, Birgitta Öberg, Leena Ala-Mursula, Jaro Karppinen, Satu Elo, Allan Abbott

**Affiliations:** 1 Unit of Physiotherapy, Department of Health, Medicine and Caring Sciences, Linköping University, Linköping, Sweden; 2 Research Unit of Health Sciences and Technology, University of Oulu, Oulu, Finland; 3 Faculty of Sport and Health Sciences, University of Jyväskylä, Jyväskylä, Finland; 4 South Savo Wellbeing Services County, Mikkeli, Finland; 5 Research Unit of Population Health, University of Oulu, Oulu, Finland; 6 Wellbeing Services County of South Karelia, Lappeenranta, Finland; 7 Wellbeing and Cultural Field of Expertise, Oulu University of Applied Sciences, Oulu, Finland; 8 Department of Orthopaedics, Linköping University Hospital, Linköping, Sweden

**Keywords:** Implementation, Occupational health services, Musculoskeletal, Qualitative, Primary care, Multiprofessional

## Abstract

**Background:**

We explored Finnish occupational healthcare professionals’ (HCP) perceptions of biopsychosocial (BPS) low back pain (LBP) management after an educational intervention.

**Methods:**

We conducted twelve group interviews of 51 physicians, physiotherapists and nurses from intervention units in a cluster randomized controlled trial (ISRCTN11875357). We used deductive and inductive content analysis to examine the data, and the Capability-Opportunity-Motivation-Behaviour (COM-B) model to identify the facilitators of and barriers to changes in three target behaviours: (A) forming a common BPS-based understanding with patients, (B) systematically using risk stratification tools, and (C) multidisciplinary collaboration in individualized care planning.

**Results:**

Facilitators and barriers were categorized into the following COM-B domains. Most of the facilitators were in the *Capability* and *Motivation* domains: increased confidence regarding managing treatment decisions, improved therapeutic alliance and renewed professional identity. Significant system-level barriers were mostly in the *Opportunity* domain: time constraints, limited resources and unclear treatment pathways. The HCPs reported improved individual skills and awareness after the training, but varying organizational policies and lacking incentives hindered the adoption of BPS methods in multidisciplinary teams. Initial resistance to change decreased as positive patient outcomes emerged. The perceived benefits were increased multidisciplinary collaboration and a shift toward holistic pain management. Those who embraced BPS management reported greater professional satisfaction and confidence when handling LBP patients.

**Conclusions:**

To effectively implement BPS management in occupational health services, organizational and system-level barriers must be addressed and HCPs’ skills and motivation enhanced. For sustained support through policy initiatives and reinforced multidisciplinary collaboration, future strategies should integrate BPS practices into routine workflows.

**Trial registration:**

The trial was retrospectively registered on 13.05.2019 ISRCTN11875357.

**Supplementary Information:**

The online version contains supplementary material available at 10.1186/s12913-025-13267-7.

## Introduction

Clinical guidelines recommend the biopsychosocial (BPS) approach for managing disabling low back pain (LBP) [1–5]. BPS management emphasizes personalized pain education, fear reduction, functional activation and the adoption of healthy lifestyle behaviours for patients for whom serious pathology has been ruled out. The intensity of the intervention should be tailored to the patient’s individual needs [6–8]. BPS interventions have produced inconsistent results. Implementing them in clinical practice has been challenging [9, 10] and optimal implementation strategies for multidisciplinary healthcare services remain unknown [11–14]. Research in the context of occupational health services (OHS) is scarce, and this highlights a crucial gap and the need for further development.

Randomized trials have repeatedly concluded that publishing clinical guidelines alone is not enough to change treatment practices [15]. Merely changing the perceptions of individual healthcare professionals (HCPs) and enhancing their professional skills through training does not necessarily lead in adopting new behaviours [16–18]. Previously identified barriers to using BPS management on LBP patients include a lack of knowledge, time constraints, traditional expectations of the physiotherapist’s role, lack of confidence and inadequate training in how to deliver BPS interventions [10, 19–22]. Implementation strategies for helping HCPs adopt BPS interventions have varied widely [11, 12, 23, 24].

A Cochrane systematic review [25] found that although multidisciplinary BPS management reduced pain and disability in persistent LBP cases, it had no similar benefits for work-related outcomes. Given that managing prolonged LBP requires a person-centred and often multidisciplinary approach, interprofessional education is essential for improving communication, clinical decision-making and care delivery [25–27]. However, research on the barriers to and facilitators of HCPs’ behavioural change in a multidisciplinary OHS setting remains limited [28–30]. This study thus addresses a critical research gap by exploring whether multidisciplinary education is perceived as an effective strategy for supporting the implementation of BPS management in OHS.

Theoretical frameworks and models based on implementation science can help us understand HCPs’ behavioural changes, evaluate implementation efforts, and identify the factors that affect implementation outcomes [26]. The COM-B (Capability, Opportunity, Motivation, Behavior) model of behaviour and the Theoretical Domains Framework (TDF) are widely used in implementation studies [31, 32]. COM-B serves as an evaluation framework for identifying the factors that influence target behaviours, and determining what needs to change to achieve the desired outcome [31]. TDF, as a determinant framework, identifies the barriers to and facilitators of implementing evidence-based behaviours [32, 33].

This study was part of a research programme involving a two-arm cluster randomized controlled trial that investigated the effectiveness, costs and implementation of guideline-oriented BPS management of LBP in comparison to routine care in Finnish OHS [34]. The intervention aimed to address the multidimensional nature of LBP and to promote a new approach to pain management in the OHS setting. The BPS training successfully shifted OHS resource use from physician-driven care (unimodal) to multidisciplinary physiotherapist-driven care (multimodal, covering more aspects of BPS) without substantial differences in total costs [35]. However, it did not significantly reduce the primary outcome of LBP-related disability during a one-year follow up [36]. To understand these results more deeply, theory-oriented and data-driven analysis approaches need to be combined. For example, implementation research frameworks can help determine HCPs’ perceptions of the facilitators of and barriers to implementing specific BPS behaviours and multidisciplinary collaboration in clinical practice.

The multifaceted implementation strategy included targeted multidisciplinary education of ‘clinical champions’ (participating physicians and physiotherapists), booster training, educational materials, outreach visits and dissemination to colleagues. The implementation object was guideline-oriented BPS management and stratified care in treatment of LBP. Target behaviours were defined on the basis of LBP guidelines, BPS recommendations, and prior research on implementation challenges [6, 10, 37].

The aim of this study was to explore how the components of the COM-B model – (1) capabilities, (2) opportunities and (3) motivation – influence the implementation of guideline-oriented BPS management of LBP in multidisciplinary OHS teams, as perceived by HCPs after a targeted educational intervention. More specifically, the study aimed to identify the barriers to and facilitators of three key BPS target behaviours: (A) HCPs and patients forming a common BPS understanding of LBP, (B) HCPs use risk stratification tools systematically at an early stage in the assessment of patients with LBP and (C) Multidisciplinary collaboration targeting an individualized plan for patients with LBP.

## Materials and methods

### Study design

The study participants were HCPs from the intervention units of a cluster randomized clinical trial (ISRCTN11875357). The trial was retrospectively registered on 13.05.2019. The physicians and physiotherapists (clinical champions)of the intervention arm received four days of training (in 2017) and three days of booster training (in 2018). The research protocol is published elsewhere [34].

**Fig. 1:**
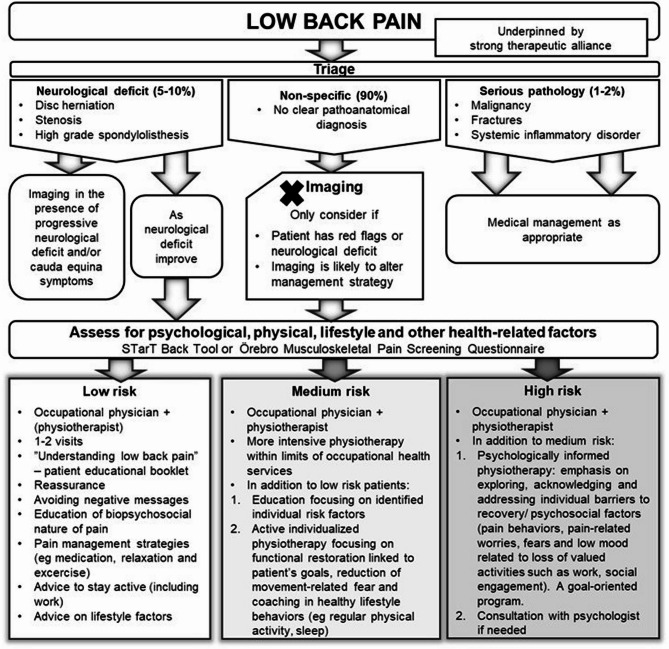
Biopsychosocial management of patients with low back pain guideline used in the study

The training emphasized a person-centred BPS approach with validating patient-professional interaction in LBP management, such as reassurance and avoiding negative messages [27]. The main risk stratification tools were the STarT Back Tool [38, 39], and the short form of the Örebro Musculoskeletal Pain Screening Questionnaire [40], which can be used to identify patients with clinically relevant psychosocial features that may delay their recovery [41–43]. A cut-off score is used to identify high-risk, moderate-risk and low-risk patient groups to enable targeted care [38, 40]. Fig. 1 shows the BPS guideline used in the study. Appendix 1 and the study protocol contain a detailed description of the educational interventions [34].

In each OHS unit of the intervention arm, the employer selected voluntary physiotherapists and physicians for training, based on their motivation and willingness to participate in an educational intervention on the BPS approach. From each OHS unit allocated to the intervention arm, at least one physician and one physiotherapist participated. The clinical champions (*n* = 28 in the initial training and *n* = 21 in the booster training; 17 HCPs participating in both sessions) from all the intervention OHS units participated in the same training intervention. The four-day training was held in September 2017 and the three-day booster training in June 2018. The clinical champions were advised to share what they learned to their OHS team members in the form of a printed educational package. To facilitate this, after the initial workshop, a research group member made outreach visits to the intervention units to provide information and to help introduce the research project to the whole workplace.

The ethics committee of the hospital district of North Ostrobothnia granted permission for the study (79/2017, 19.9.2017). The study was conducted in accordance with the Declaration of Helsinki. The University of Oulu acted as the research registrar. We followed the consolidated criteria for reporting qualitative research (COREQ) when reporting the results [44]. All personal details were replaced by identification codes to ensure the participants’ full anonymity. The research team has contributed to multiple qualitative studies in the context of healthcare and professional education that have been published in peer-reviewed journals. The team acknowledges that their cultural and theoretical backgrounds may have influenced the interpretation of the data. The first author identifies as a middle-class woman, and was trained to deliver the BPS approach in physiotherapy during the previous study [45], and this informed her understanding of how to implement the BPS management of LBP. She approached the study from a constructivist approach, assuming that reality is socially constructed, and her task as a researcher was to understand and interpret socially constructed meanings.

### Setting

Five nationwide OHS organizations participated in the study. Finnish OHS is a key healthcare provider for the working population, and handles both work-related and general health issues, including acute and chronic illnesses. OHS operates through collaboration between healthcare providers and workplaces, and focuses on occupational safety and maintaining work ability. This focus integrates primary care with preventive functions, meaning that work-relatedness and work ability are evaluated during appointments. The multidisciplinary OHS team, which consists of physicians, nurses, physiotherapists and psychologists, works together to deliver comprehensive care [46] through regular interaction and collaboration, and values the expertise of various HCPs [47].

We conducted focus group interviews (*n* = 12) (Table 1). All the clinical champions and their multidisciplinary OHS teams who treated LBP patients were invited by their employers to participate in the interviews. Their participation was voluntary, and they received no compensation from the research team. The OHS employers kept no record of those who refused to participate. Informed consent to participate was obtained from all the study participants.

### Description of sample

The participants represented a purposive sample of HCPs (*n* = 51) working in intervention units. They were from all over Finland and worked in both private (*n* = 42) and public (*n* = 8) OHS units. Eleven of the participants were male and 40 were female. They were physicians (*n* = 20), physiotherapists (*n* = 24) and nurses (*n* = 7). We managed to interview the majority of the clinical champions (25 of 28) and their multidisciplinary team members (*n* = 26) including the head physicians, head nurses and/or head physiotherapists in each OHS company. No psychologists were interviewed.

**Table 1:** Focus group interviewees

Unit code	Number of focus group interviews	Number of participants	Number of occupational healthcare professionals		
			Physicians	Physiotherapists	Nurses
PC #1	3	6	3	2	1
PC #2	4	26	10	10	6
PC #3	1	2	1	1	0
PC #4	2	9	3	6	0
PuC #2	2	8	3	5	0
Total (n)	12	51	20	24	7

### Data collection

We created a semi-structured interview guide to cover the target behaviours of the BPS approach (Appendix 2). The participants were also encouraged to speak freely about their participation in the study and the management of LBP patients in OHS. A practice interview was conducted with a physiotherapist who was not involved in this study but had undergone BPS training. The practice interview helped us clarify and order the interview questions. The multidisciplinary OHS teams were interviewed in the spring of 2019, either in the OHS units or in some other agreed upon way (e.g. remote meeting). The interviewer (MP) had a Masters’ degree in health care, was a member of the research group and worked as a physiotherapist at the time of the study. She was present during the educational interventions but was otherwise unknown to the participants. The interviews were conducted in Finnish. In three of the interviews, a research assistant was present to assist with the data collection by making the audio-visual recordings.

The interviews lasted 59 min on average (28–79 min). They were audio- and videorecorded and the first author transcribed them verbatim. The resulting data consisted of 281 pages (font = Times New Roman 12, spacing = 1.5). The interviewer made field notes during and after each focus group session to document the required contextual information. We did not carry out participant validation of the transcripts or repeat any interviews.

### Data analysis

We combined deductive and inductive approaches for analysing the data. The analysis process contained a preparation phase, an organizing phase, a reporting phase, and a results phase, as described by Elo and Kyngäs [48]. In the preparation phase, the participants’ statements were read and re-read to gain familiarity. The unit of analysis was a set of ideas selected from the focus group data and could be one sentence or several sentences. In the deductive organizing phase, we developed a two-level categorizing matrix (Appendix 3), and the first author coded the data according to these categories. The original statements from the interview data were first coded using a deductive, i.e. theory-oriented, method. In Step 1, the coded statements (*n* = 1546) were classified into three target behaviours, 14 TDF domains [32] and the COM-B model [31] and interpreted as possible facilitators of or barriers to the achieving target behaviours. We used data analysis programme MAXQDA 2020 Analytics Pro to manage the data.

Each matrix category underwent inductive analysis. MP and RH used the principles of inductive content analysis to develop the categories in each TDF domain [48]. In Step 2, we extracted all the expressions that corresponded to the unit of analysis that addressed the research question from the material and coded them. In Step 3, we formed the subcategories by combining the coded expressions and deciding what belonged in the same categories. We named the categories using the expressions that most representatively described them. Next, the subcategories were conceptualized and abstracted into a generic category. Table 2 shows an example of the analysis and categorization process. All the authors had the opportunity to participate in discussions, provide feedback and agree on the final categories. Finally, we combined the identified facilitators and barriers into a generic category for reporting the results and comparing them to those of previous studies.

**Table 2:** Example of deductive and inductive analyses and categorization process

Target behaviour A. HCP and patient form a common individual BPS understanding of LBP				
**Step 1 Deductive content analysis** Data reviewed for content and coded for correspondence with or exemplification of target behaviour and TDF domain		**Step 2** **Inductive content analysis**	**Step 3 Inductive content analysis** Conceptualizing and abstracting into categories	
COM-B/ TDF domain (description)	Example of quotation	OPEN-CODE	SUB-CATEGORY	GENERIC CATEGORY
MOTIVATION/Social and professional role, identity (Interpersonal processes that can cause individuals to change their thoughts, feelings, or behaviours)	*‘This has somehow broadened my awareness*,* and I no longer pay attention to only the mechanics*,* instead I consider functional movements and whether there is that pain-related fear and what causes it.’ (OPT*,* 3 days training; PC4)*	Role of OPT developed from bio to BPS	Expanding professional role, confidence and boundaries	Renewing professional identity
	*‘I invest quite a lot in such dialogical discussion*,* much more than before. Previously*,* it was more about advising and taking control of the situation*,* almost like lecturing the client. Now it’s more of a biopsychosocial approach with dialogue*,* which is good.’ (OPT*,* 7 days training; PC2)*	Role of OPT developed from lecturing to person-centred		
	*‘I give a certain amount of guidance in basic things to these people with acute back pain*,* who don’t seem to need the guidance of a physiotherapist at the beginning… acute pain phase self-care methods*,* relaxation techniques*,* and then walking*,* how to start*,* and gradually increase activity as the pain allows*,* those I always go through ‘(OHP*,* 7 days training; PC1)*	OHP starts giving BPS patient education		
	*‘And because we’ll always refer our patients to physiotherapy whenever they are in pain*,* and we wait for the professionals to give them guidance and then see how rehabilitation has progressed.’ (OHP*,* 4 days training; PC2)*	Passive professional role: OHPs’ role is to follow progress of rehabilitation	Remaining in familiar professional role and within boundaries	
	*‘I haven’t gained much from this. Because*,* of course*,* I’ve been a physician for such a long time.’ (OHP*,* team member; PC2)*	Familiar professional role		
	*‘Yes*,* through training*,* I’d say that it has somewhat changed the entire patient interaction from my side*,* trying to understand the client’s own thoughts and what the client themselves sees as causing their issues.’ (OHP*,* 7 days training; PC1)*	Changing approach to encountering all patients	Developing identity as a professional	
	*‘I guess I listen more and then the treatment is more individualized. And it produces results. I’m still learning in my old age. This dialogue approach applies even in the company of friends.’ (OPT*,* 7 days training; PC2)*	HCP perceives benefits of utilizing validation skills in their personal life		

*COM-B* Capabilities, Opportunities, Motivations, Behaviour model, *TDF* Theoretical Domains Framework, *BPS* biopsychosocial, *OHP* occupational health physician, *OPT* occupational health physiotherapist, *HCP* healthcare professional, *LBP* Low back pain

## Results

**Fig. 2:**
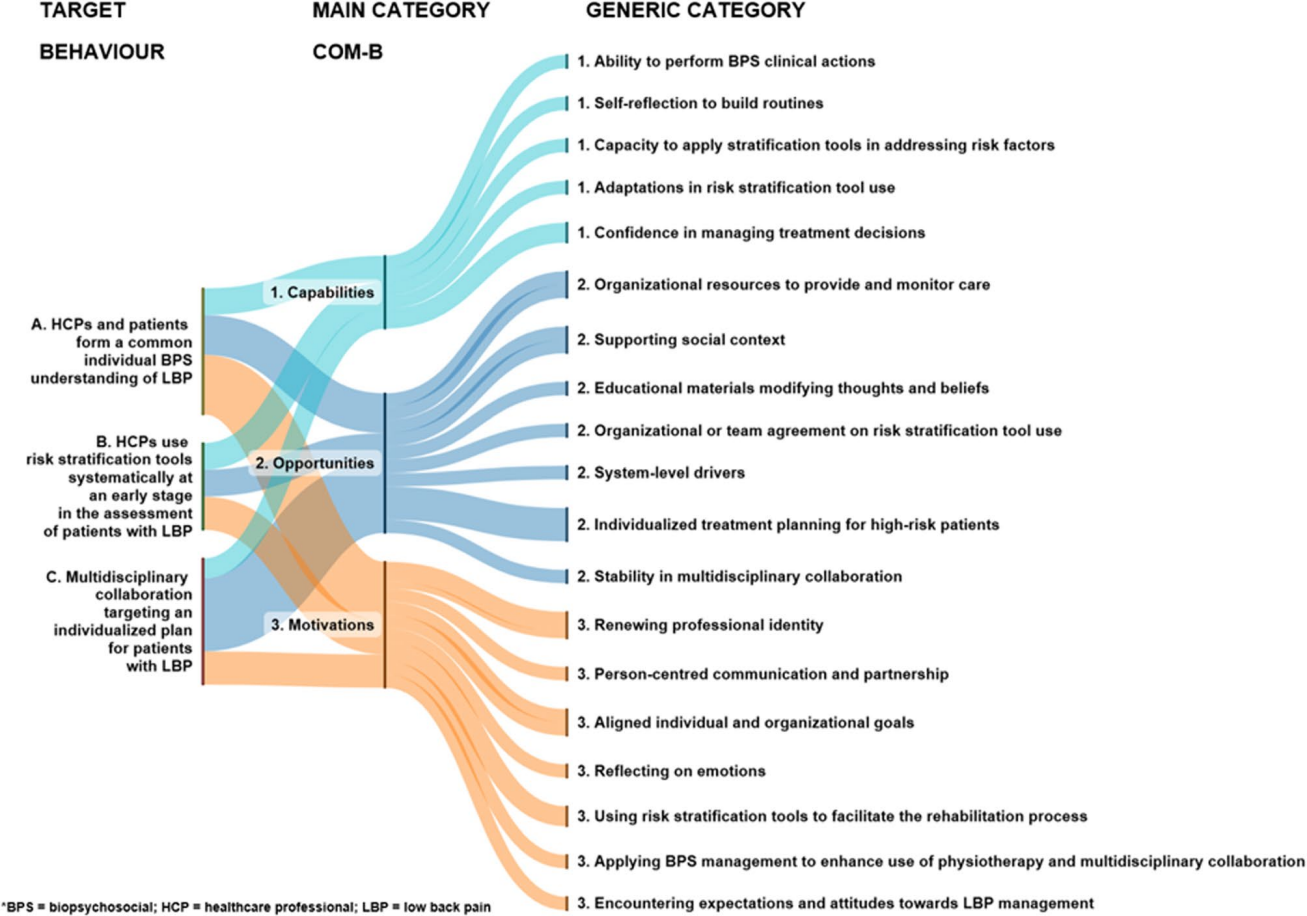
Summary of capabilities, opportunities, and motivations for implementing guideline-oriented biopsychosocial management of low back pain

We identified a total of 19 TDF-related categories that represented the capabilities, opportunities and motivation for implementing guideline-oriented BPS management of LBP. The following figure presents the main and generic categories (Fig. 2).

**Fig. 3:**
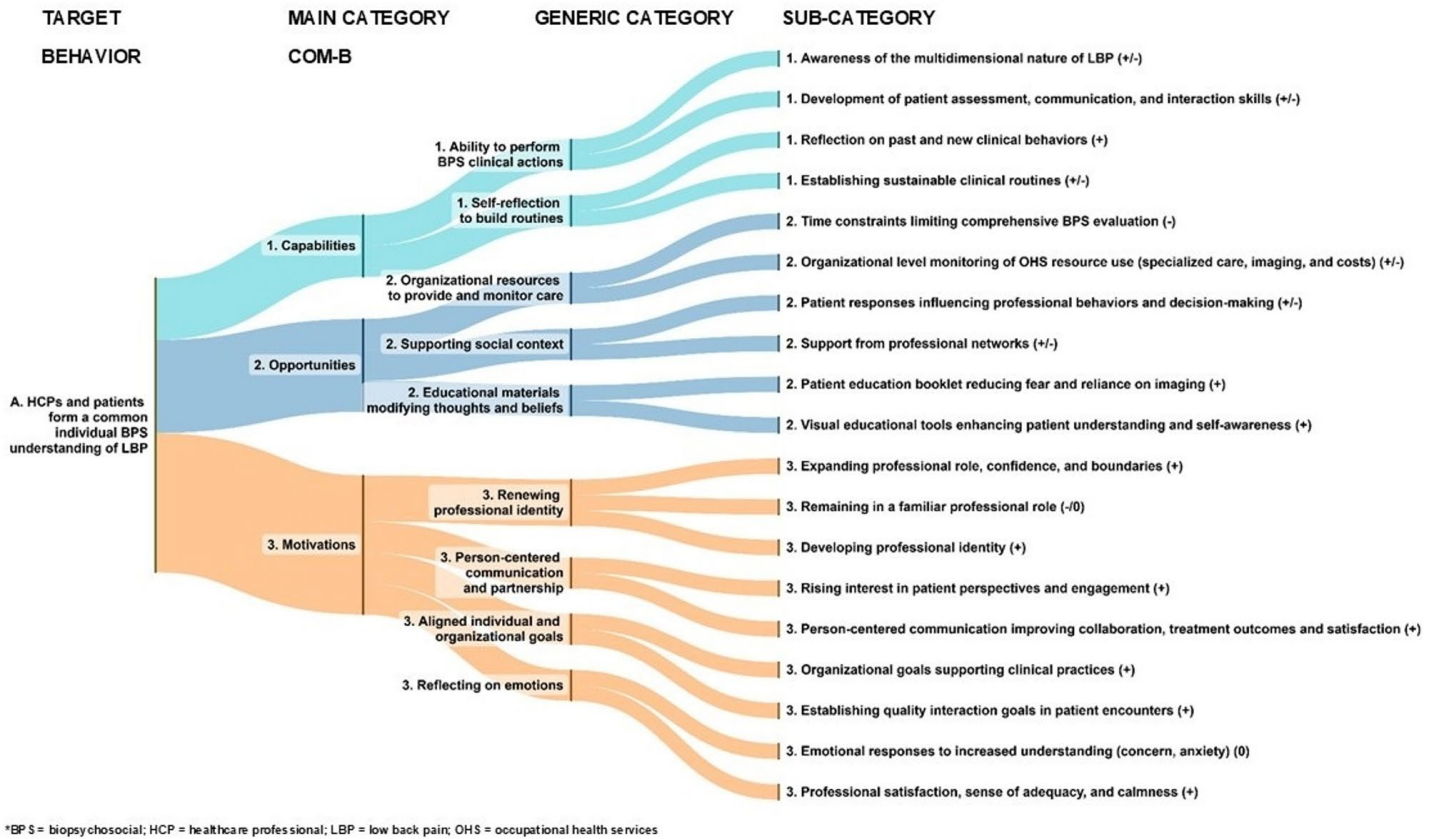
Healthcare professionals and patients form a common individual biopsychosocial understanding of low back pain

**Fig. 4:**
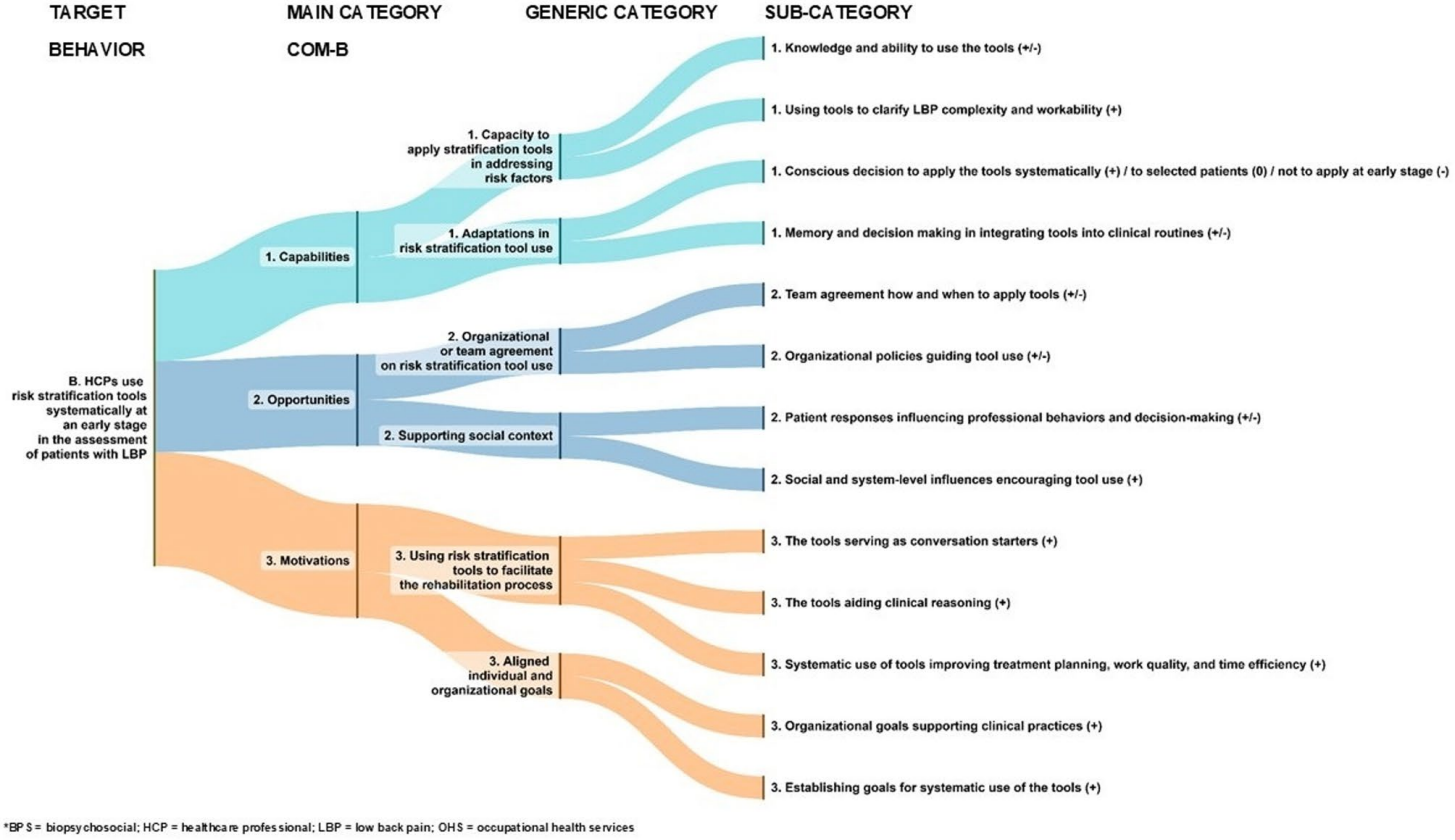
Healthcare professionals systematically use risk stratification tools during patient assessment

**Fig. 5:**
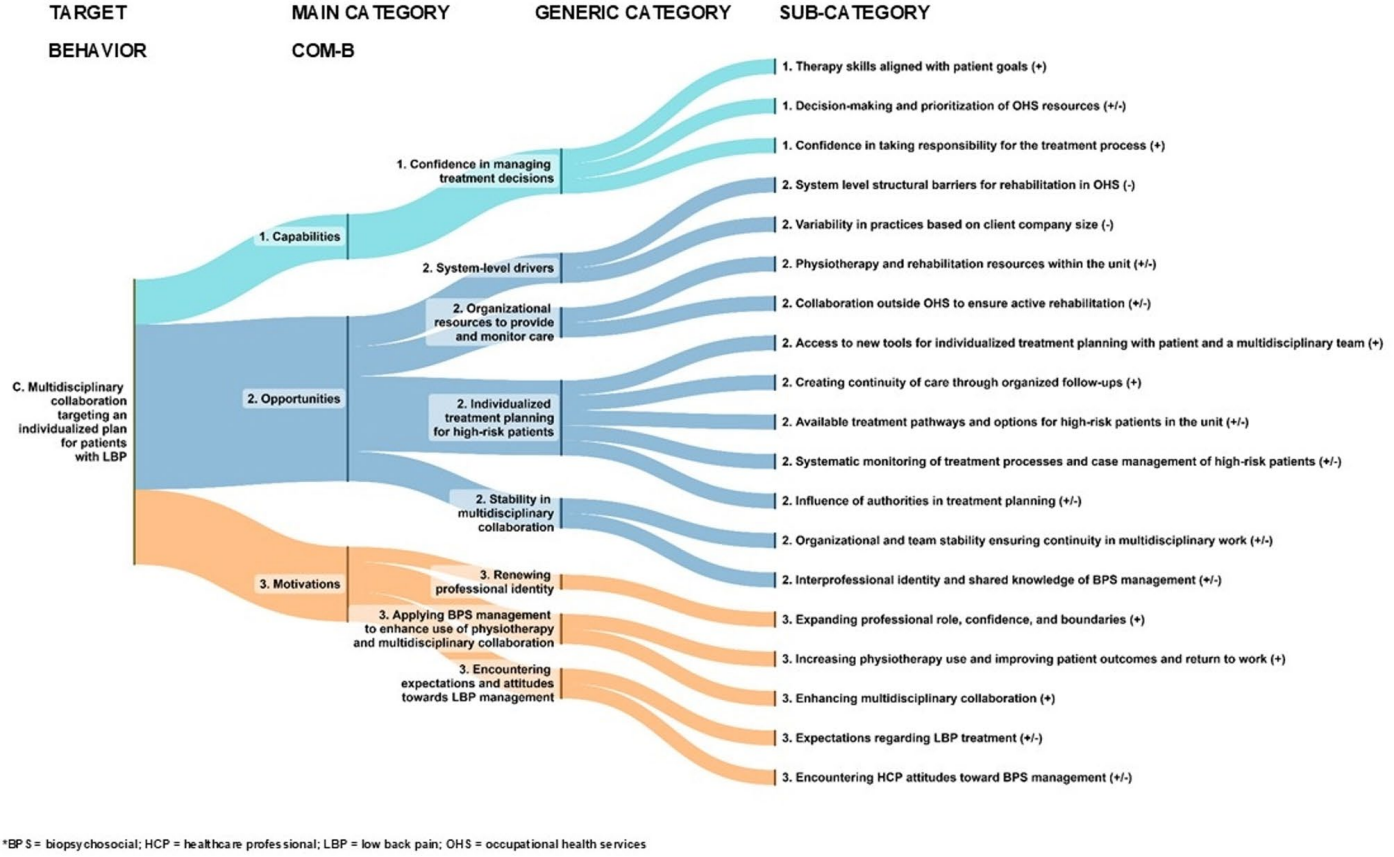
Multidisciplinary collaboration targeting an individualized plan for patients with LBP

Next, we present a detailed analysis of the study findings, focusing on the identified facilitators of (+), barriers (-) or neutrals (0) to achieving the three BPS target behaviours. Figures 3, 4 and 5 illustrate the inductive categories (generic and subcategory) under three main COM-B categories for each target behaviour. More detailed information on the main, generic and subcategories are provided in Supplementary Tables 1a–c, and quotations are presented in the Online material.

## Target behaviour A

### HCPs and patients form a common individual BPS understanding of LBP (Fig. 3)

#### 1) Capabilities of HCPs to form a common individual BPS understanding of LBP with patient

##### Category – Ability to perform BPS clinical actions (+/-)

Facilitators: The HCPs perceived that their understanding of and skills for managing LBP patients had improved, and that they had developed a deeper understanding of the multidimensional nature of pain and of the impact of communication on patient recovery. Considering patients’ life situations and recognizing pain-related fear of physical activity were seen as essential components of BPS assessment. The physiotherapists felt more confident about rationalizing the need for imaging and felt empowered to discuss issues they previously considered beyond their competence. Most of the HCPs also felt more confident about encouraging patients to use their backs.

Barriers: The multidisciplinary team members perceived their knowledge of and skills in BPS management as superficial when applied in clinical practice.

##### Category – Self-reflection to build routines (+/-)

Facilitators: To implement new clinical behaviours, the HCPs felt that they needed to self-reflect on and be critical of their previous practices, such as recognizing invalidating communication habits and challenging themselves to actively develop new routines. Other facilitators were identifying previously missing skills and fostering patients’ self-efficacy and hope. Many team members reported feeling more confident about reinforcing the importance of staying active and engaged in work after being inspired by discussions with clinical champions. The HCPs perceived that this shift was possible because they no longer feared the patient’s pain and felt more confident about normalizing pain in clinical discussions and actions. This deeper understanding was reflected in patient assessments, as the HCPs reported spending more time interviewing and listening and allowing patients to express their emotions. The physiotherapists in particular reported successfully implementing multidimensional assessments during their allocated times by consciously prioritizing patient interviews over performing an extensive range of clinical tests.

Barriers: The HCPs perceived that they needed to reorganize their work to integrate BPS management as a natural part of their routine practice. However, difficulty finding alternative ways of working and a lack of time for processing new knowledge were perceived as barriers.

#### 2) Opportunities to form a common individual BPS understanding of LBP with patient

##### Category – Organizational resources to provide and monitor care (+/-)

Facilitators: Organizational-level directives and the monitoring of the use of resources (e.g. referrals to physiotherapists, specialized care, imaging and care-related costs) were perceived as facilitators. In one organization, BPS assessment of LBP patients was prioritized by a directive to allocate 30 min for a patient’s first physicians’ appointment, which allowed more time for a comprehensive assessment.

Barriers: Many of the physicians perceived insufficient consultation time as a barrier to conducting a multidimensional BPS assessment. They often avoided addressing complex issues if the appointment was too short to adequately help their distressed patients. When patients independently chose to pay for imaging themselves, it was perceived as difficult to establish a shared understanding of BPS management.

##### Category – Supporting social context (+/-)

Facilitators: The HCPs perceived that they had become more responsive to patients’ experiences and expectations, as well as more understanding. Patients’ responses were seen as shaping HCPs’ behaviours and decision-making, and many reported receiving positive feedback on the multidimensional pain explanation. They also regarded professional networks and training as essential for supporting the transition to BPS clinical behaviours.

Barriers: Lack of support from colleagues, contradictory instructions and explanations given to patients by different HCPs, and patients’ negative expectations, such as a strong desire to retire, were perceived as barriers to implementation.

##### Category – Educational materials modifying thoughts and beliefs (+)

Facilitators: Having access to educational materials that addressed beliefs, fears, understanding and self-awareness was perceived as a facilitator. These resources helped provoke changes in patients’ thinking patterns, enabling them to reframe their perceptions and gain new insights. The patient education booklet was valued for its role in managing patients’ fears and reducing the need for imaging. The physiotherapists found that the booklet enhanced their credibility when discussing imaging needs, which raised the threshold to referring the patient to a physician for imaging. The physiotherapists also described using resources such as Explain Pain videos and video recordings made during appointments to help patients recognize the disconnect between their thoughts and behaviours. These tools were perceived as facilitating a shared understanding between HCPs and patients.

#### 3) Motivations to form a common individual BPS understanding of LBP with patient

##### Category – Renewing professional identity (+/-/0)

Facilitators: These included physicians’ and physiotherapists’ perceptions of their expanded social and professional roles. Many physicians reported that they had begun guiding patients with acute LBP on self-care methods, relaxation, movement and a gradual return to activity. Some HCPs perceived that learning BPS management had completely changed their approach to patient care. The physiotherapists even reported applying these new interaction skills outside of work in their personal lives. This showed that these skills had become routine – something they naturally incorporated and benefitted from in their daily interactions.

Barriers/Neutrals: Many team members perceived that BPS management was already integrated into their professional roles. A barrier to some physicians was their perceived passive role in the rehabilitation process. Patients referred to physiotherapy expected the physiotherapists to provide the necessary guidance, whereas the physician’s role was primarily to monitor the progress of rehabilitation. These physicians perceived their professional role as unchanged.

##### Category – Person-centred communication and partnership (+)

Facilitators: Person-centred communication was perceived as improving therapeutic alliance, collaboration, treatment results and patient satisfaction. The HCPs discovered a new interest in their patients’ perspective and understanding. They found that letting the patient be the storyteller was an important step for forming a common BPS-based understanding with the patient and for finding goals for meaningful rehabilitation from the patients’ perspective.

##### Category – Aligned individual and organizational goals (+)

Facilitators: The HCPs reported setting individual goals to focus on the quality of their interaction with the patient (e.g. nocebo messages, plain language, shared understanding). An organizational endeavour to apply BPS management was also perceived as facilitating its adoption on an individual level. In one organization, all the physiotherapists compared the benefits and challenges of BPS management to their normal practices. This strategy helped dismantle misconceptions and facilitated acceptance and willingness to use BPS management as the first-line treatment in OHS. In addition, recognizing that the organization’s goals and current development models (work modification, care pathways and reducing sick leaves) were in line with the BPS approach encouraged managerial support of changes in clinical practices.

##### Category – Reflecting on emotions (+/0)

Neutrals/Facilitators: The HCPs reported feeling more worried than before about patients’ fear avoidance behaviour and feeling anxious when patients misunderstood them. These were categorized as neutral, because the HCPs believed this concern and anxiety was a response to their increased understanding. Despite this, the HCPs perceived that the new way of working increased their satisfaction, feeling of adequacy and calmness during patient encounters.

## Target behaviour B

### HCPs use risk stratification tools systematically at an early stage in the assessment of patients with LBP (Fig. 4)

#### 1) Capabilities for the systematic use of risk stratification tools at an early stage in the assessment of patients with LBP

##### Category – Capacity to apply stratification tools in addressing risk factors (+/-)

Facilitators: These included an improved ability to identify patients’ psychosocial profiles with the help of tools. The tools made it easier to assess and address risk factors, and reduced the complexity of pain problems or work disability.

Barriers: Some team members perceived their lack of knowledge about the stratified model of care as a barrier, which suggests that dissemination might not have been completely successful in some intervention units.

##### Category – Adaptations in risk stratification tool use (+/-/0)

Facilitators: Some HCPs reported using risk stratification tools systematically for assessing work ability. Having a clear process for using the tools – during appointments or as homework – and documenting the scores in the electronic patient records were perceived as facilitating the tools’ use in clinical practice.

Neutrals: The most common way in which the tools were used differed from their original intended purpose. Instead of systematic use, the HCPs reported typically using them for selected patient groups, such as new patients or those with persistent pain problems. The HCPs perceived the choice of a questionnaire as personal, given the wide variety of available assessment tools.

Barriers: Barriers to applying the tools as intended included HCPs’ perceptions that patients needed time to build up trust before bringing up psychosocial issues in an interview. A few HCPs found the tools overly complex or felt that they focused too much on psychological aspects and considered other assessments more important. Some physicians prioritized the assessment of work ability and the need for medication over other considerations, particularly in the context of limited time resources.

#### 2) Opportunities to systematically use risk stratification tools at an early stage in the assessment of patients with LBP

##### Category – Organizational or team agreement on risk stratification tool use (+/-)

Facilitators: A facilitator of the systematic use of questionnaires was their availability in electronic form. Some teams perceived that discussions on tool usage and task division among professionals helped them reach clear agreements on when to use questionnaires systematically, such as in work ability assessments.

Barriers: The HCPs felt that their intention to use the tools was hindered by the lack of a mutually agreed practice at the organizational level, and their limited ability to influence organizational policies.

##### Category – Supporting social context (+/-)

Facilitators: System-level and social pressure encouraged the HCPs to use the tools. For example, physical and rehabilitation medicine specialists required a comprehensive evaluation, also of psychosocial factors, before consultation, and insurance companies preferred work ability risk assessments to be a part of the decision-making process.

Barriers: Some HCPs perceived negative patient responses to the tools as a barrier, leading them to discontinue their use.

#### 3) Motivations for systematically using risk stratification tools at an early stage in the assessment of patients with LBP

##### Category – Using risk stratification tools to facilitate the rehabilitation process (+)

Facilitators: Many HCPs emphasized how the tools helped them start the rehabilitation process by aiding conversation. The physiotherapists noted that the questionnaire prompted them to consider the patient’s broader life situation. The HCPs found that using the tool as intended supported clinical reasoning and enabled them to develop more individualized treatment plans. The systematic surveying of psychosocial factors was perceived as enhancing the quality of OHS care.

##### Category – Aligned individual and organizational goals (+)

Facilitators: Many HCPs had set a goal to integrate the tools into their practice. Some organizations planned to incorporate them into their electronic system, allowing patients to complete the questionnaire before their appointment so that the results were available at the first consultation. The digitalization of risk stratification tools was seen as an opportunity to guide the direction and extensiveness of the rehabilitation process. The organization’s goal of using the questionnaire for every LBP patient, along with training for professionals, was perceived as a facilitator for systematic use, even though this was not yet fully implemented.

## Target behaviour C

### Multidisciplinary collaboration targeting an individualized plan for patients with LBP (Fig. 5)

#### 1) Capabilities for multidisciplinary collaboration targeting an individualized plan for patients with LBP

##### Category – Confidence in managing treatment decisions (+/-)

Facilitators: The physiotherapists felt that the BPS training had improved their confidence in their therapy skills and in their ability to treat more challenging patients. This included integrating patients’ individual goals into treatment plans and focusing more on functional movements in daily activities. Many team members, including physicians and nurses, reported increased trust in the multidisciplinary team’s capabilities. The physicians felt that they had gained confidence in taking responsibility for the treatment process and developed patience to wait for rehabilitation results. This shift resulted from a conscious decision to utilize all the multidisciplinary resources available in the unit before referring patients to specialized care or imaging.

Barriers: Some physiotherapists felt like outsiders in the multidisciplinary teams, as they were excluded from discussions and not involved in decision-making regarding the treatment and rehabilitation process of LBP patients, including the use of multidisciplinary OHS resources.

#### 2) Opportunities for multidisciplinary collaboration targeting an individualized plan for patients with LBP

##### Category – System-level drivers (-)

Barriers: Many HCPs reported system-level structural barriers to early-stage rehabilitation. For example, at the time of the study, Finnish OHS policy restricted patients to a maximum of three physiotherapy appointments. Variations in practices based on the size of the client companies were also seen as barriers to multidisciplinary collaboration and treatment planning. The lack of formal communication and team discussions stemmed from the absence of a payer for structured multidisciplinary meetings on patient care, a challenge particularly evident in small client companies. In contrast, the rigid practices of large organizations limited opportunities in local OHS.

##### Category – Organizational resources to provide and monitor care (+/-)

Facilitators: Direct access to physiotherapy within one to three days and the flexibility to use physiotherapy based on patient needs were seen as facilitators.

Barriers: The perceived barriers to resources in the OHS unit included long waiting times for physiotherapy and insufficient availability of BPS-trained physiotherapists. When patient treatment required healthcare services outside of OHS, many HCPs felt they lacked personnel resources to monitor rehabilitation processes and were unable to influence the content of the rehabilitation.

##### Category – Individualized treatment planning for high-risk patients (+/-)

Facilitators: Using risk stratification tools was perceived as a facilitator of individualized patient treatment planning in the OHS multidisciplinary team. The Patient-specific Functional Scale was also considered a valuable tool for identifying functional goals together with the patient, helping set meaningful goals for both leisure time and work. The physiotherapists viewed an authoritative physician as helpful for convincing patients of the importance of their treatment plan. The HCPs supported treatment planning by ensuring continuity of care through organized follow ups, such as phoning patients two weeks after their physiotherapist appointments so that they were not left without support. Systematic monitoring of treatment processes and sick leaves, and case management of high-risk patients were facilitated by team-agreed task allocation. In some OHS units, the HCPs had developed innovative BPS-based service models, such as multidisciplinary group rehabilitation, and rapid multidisciplinary collaboration for high-risk patients.

Barriers: The availability of care pathways for high-risk patients was perceived as varying greatly. The physiotherapists identified the lack of collaboration with psychologists for LBP patients as a barrier. Non-individualized treatment plans from orthopaedic surgeons were also considered unhelpful for BPS management. Many HCPs concluded that LBP care pathways were either unclear or non-existent.

##### Category – Stability in multidisciplinary collaboration (+/-)

Facilitators: Stability in the organization was perceived as ensuring the continuity of multidisciplinary work. Regular interprofessional meetings ensured that knowledge transfer was not solely based on electronic patient records. Many HCPs felt that after the multidisciplinary training they more often discussed patients’ situations and planned treatment processes in OHS team meetings. In some OHS units, the clinical champions developed a group identity and shared similar thoughts on how treatment should be carried out. Interprofessionally shared knowledge of how to use BPS management was perceived as facilitating sustainability, and as unifying and harmonizing care pathways.

Barriers: These included changes to job descriptions, organizational reforms, digitalization, lack of time, financial constraints, high staff turnover and short work periods. These factors were perceived as hindering multidisciplinary collaboration and implementation efforts. Some clinical champions found it difficult to disseminate BPS management within multidisciplinary OHS teams due to a disconnect between BPS training and routine teamwork. Physicians in particular found leading implementation challenging due to their limited connection to patient care, having to rely on electronic patient records for transferring knowledge, and the large size of their units, which made effective implementation difficult. A key barrier was the perception that the BPS approach should be personally internalized and embraced for successful adoption. In addition, the professionals’ actions during patient encounters could not be directly monitored or controlled, which further complicated dissemination.

#### 3) Motivations for multidisciplinary collaboration targeting an individualized plan for patients with LBP

##### Category – Renewing professional identity (+)

Facilitators: The physiotherapists felt that their professional skills had expanded as their role in OHS was no longer limited to physical ergonomics. Many reported gaining confidence in applying their expertise more broadly in multidisciplinary collaboration. The HCPs observed positive patient experiences with BPS management in both mental and physical conditions. The physicians recognized the increased role of physiotherapists in addressing pain behaviours, fears and treatment. The patients’ feedback to the HCPs revealed that they perceived physiotherapists as appropriate providers of psychological support, often finding their guidance sufficient without the need for additional psychological intervention.

##### Category - Applying BPS management to enhance use of physiotherapy and multidisciplinary collaboration (+)

Facilitators: The HCPs perceived that using BPS management led to increased use of physiotherapy, improved patient outcomes, including return to work and less referrals to specialized care. It was also seen as enhancing multidisciplinary collaboration by promoting consultations and interprofessional referrals. The HCPs also found multidisciplinary collaboration more effective than before.

##### Category – Encountering expectations and attitudes towards LBP management (+/-)

Facilitators: These included enthusiasm for a new approach to patients and a shared commitment to identifying their abilities and opportunities despite LBP. The physiotherapists found it helpful that patients generally expected to receive instructions and advice when they were asked about their expectations, as this made it easier to engage them in active treatment.

Barriers: The HCPs saw patients’ expectations to be referred to physiotherapy for passive treatments as a barrier. Some HCPs also relied on imaging to reinforce patient commitment to rehabilitation and were sceptical of BPS-based care. Many clinical champions reported initial resistance from OHS team members, who displayed low enthusiasm, disbelief or frustration toward BPS management. However, these attitudes gradually improved as the team members observed successful patient outcomes and shared patient experiences. As a result, most HCPs expressed an interest in further BPS training for their work community.

## Discussion

The aim of this study was to explore the perceptions of HCPs, including clinical champions and their multidisciplinary team members, regarding the capabilities, opportunities and motivations for implementing guideline-oriented BPS management of LBP after a targeted educational intervention. Using a combination of deductive and inductive analyses of interviews with HCPs, we identified both barriers to and facilitators of BPS management in all the COM-B model categories one year after it was first implemented. The barriers were predominantly in the opportunity domain and mainly at the organizational level, whereas the facilitators were in the capability and motivation domains.

### Capability

Systematic reviews indicate [11, 49] that successful HCP behaviour change interventions typically involve face-to-face training over an extended period of one or more months, covering patient demonstrations, pragmatic tools and opportunities to practice new skills in clinical settings. The trainers’ feedback also plays a critical role in reinforcing learning [11]. In the present study, multidisciplinary training was perceived as enhancing the HCPs’ confidence in both their own and their multidisciplinary team’s abilities to manage complex patients, creating trust in each other’s capabilities and optimism that this would lead to positive outcomes for the patient.

The HCPs acknowledged that creating awareness and shared views on BPS management both within their teams and with patients was challenging. A common understanding of the multidimensional nature of pain, the use of relevant clinical tools and interprofessional roles facilitated the implementation of BPS management in OHS. The HCPs saw clinical routines that are collaboratively accepted by the multidisciplinary team as important facilitators.

The challenges of shifting from a biomedical to a BPS-based approach in LBP management are well-documented in several qualitative studies [10, 18, 19, 22, 50, 51]. Traditionally, physiotherapists focus on the somatic aspects of pain, and pay insufficient attention to the psychological and social dimensions [18, 52, 53]. Previous studies indicate that physiotherapists may avoid addressing emotionally charged topics, which can lead to invalidating communication and limiting patients’ expression [54]. However, brief validation training can improve HCP communication skills [55, 56]. The findings of this study support this, as the HCPs felt that the tools helped them address BPS issues, communicate more effectively with patients, and encouraged patient expression of emotions.

Behaviour change techniques linked to the TDF [57–59] emphasize the importance of instructions, graded tasks and active problem solving, such as analysing the factors that influence behaviour and generating strategies to overcome barriers and/or increase facilitators, to enhance skills and self-efficacy. Establishing a method for the HCPs to monitor their behaviours could also be part of a behaviour change support strategy [57–59]. In this study, memory and decision-making regarding the use of the tools in clinical routines were often perceived as barriers. These could be counteracted by defining a stimulus to cue the behaviour [57–59], such as a prompt in the electronic patient information system to design a care pathway for high-risk patients.

### Opportunity

The HCPs described how multidisciplinary collaboration was strengthened by the social influence in the teams, which provided support and fostered the stability and continuity of care. One year after its implementation, BPS management of LBP, including risk stratification, had not been fully adopted in the OHS units. This may partly explain the lack of significant differences between the patient outcomes in the intervention and control arms [36].

Implementation research suggests that behaviour change occurs at the level of the work community as a whole [60] and requires shifts in perceptions, beliefs and clinical routines across individual, group and organizational levels [61, 62]. Such changes often take time, and require HCP involvement in the development of clinical procedures [61, 62] as well as teamwork availability [63]. This study reinforced these findings, as the HCPs reported limited organizational and work community-level changes.

One notable barrier was the lack of clear pathways between OHS and primary care. Previous studies have identified time constraints and remuneration issues as barriers to delivering risk-stratified care in primary care [51, 64–66]. In this study, organizational structures and resources were sometimes inadequate to ensure the continuity of care for high-risk patients. In the absence of structured care pathways, the HCPs attempted to manage them individually. The facilitators in this study included monitoring care pathways, coordinating multidisciplinary resources, and incorporating group-based treatments for high-risk patients.

Environmental contexts and available resources significantly influence HCPs’ behaviour. Practical support from colleagues or staff, modifications to daily routines and social reinforcement (e.g. recognition of effort) can facilitate change [57–59]. Monitoring clinical behaviours and providing feedback could be a key task for clinical champions, and may facilitate sustainable implementation in clinics [67]. In our study, the clinical champions created opportunities for their colleagues to use BPS management by disseminating their knowledge. Open discussion on norms and agreement on clinical practices was perceived as facilitating the adoption of BPS management.

### Motivation

The COM-B model [31] recognizes that motivation is a key driver of behaviour change. One qualitative study [45] reported considerable initial resistance to BPS management among Finnish physiotherapists because the approach was new and different. Learning and implementing BPS management without adequate support was perceived as challenging. In our study, the HCPs encountered both enthusiasm and resistance in their multidisciplinary teams. Attitudes towards BPS clinical behaviours were influenced by the credibility of the information sources advocating for or against its adoption. Identifying oneself as a role model when acting as a clinical champion and that one’s behaviour might serve as an example to others was sometimes unclear. On the other hand, the teams with multiple trained clinical champions demonstrated greater enthusiasm and engagement, indicating that multidisciplinary training was a valuable implementation strategy for enhancing collaboration and supporting the adoption of BPS management.

Qualitative studies of physiotherapy norms have highlighted that professional identity is shaped by educational and institutional expectations, often reinforcing a biological focus on pain management [68, 69]. In our study, a *Renewing professional identity* category emerged in two target behaviours (A, C). For some, their professional role expanded to align with BPS principles, whereas for others their old roles remained the same, and they saw no difference between their current and the expected behaviours. As a behavioural change strategy, beliefs about capabilities can be enhanced through demonstration, role modelling, and providing opportunities for behavioural practice and feedback. Multidisciplinary reflection and mutual learning require dedicated time during the workday [70] the lack of which, due to organizational constraints, was identified as a major barrier in our study.

Goal setting, at both the individual and organizational level, was perceived as facilitating engagement with BPS management (A, B). Open discussions on implementation goals, with comparisons of the benefits and challenges of behaviour change, were perceived as facilitators. One participating organization adopted this strategy, and its physiotherapists found that such discussions clarified misconceptions. Although the literature identifies discussing goals and benefits as an effective strategy for supporting behaviour change [57–59, 71], reinforcement strategies may also include material incentives or rewards [57–59]. However, this study did not recognize possible reimbursements.

HCPs play a critical role in coaching, educating and empowering patients to manage their conditions independently. Empathy and validation in clinical interactions enhance patient engagement and adherence, leading to improved patient outcomes [72–76]. Interestingly, the HCPs in this study reported strong emotional responses when reflecting on patient encounters. Some described professional satisfaction and unity within their multidisciplinary teams, but others experienced anxiety and concern as a result of their increased awareness and understanding of patient perspectives. Although self-reflection is discussed in the literature, the dual focus on emotional reflection and its connection to renewing professional identity in the context of BPS implementation may signal deeper professional and identity transformations, an area that is important to address in future implementation strategies.

### Strengths and limitations of the study

The focus group interviews were conducted one year after the educational intervention and booster training, due to the requirement for extended follow-up in multidisciplinary implementation studies [70, 77, 78]. A further strength was that the implementation strategy was in line with conventional continuing education models. However, the relatively short training (3–7 days) and lack of long-term support is likely to have limited any behavioural changes. More intensive training, mentoring and organizational support could improve skills and the willingness to provide BPS care and conditions for HCPs to transfer their knowledge to clinical practice in their own OHS units and potentially even across organizations. Clinician beliefs and attitudes have most probably reflected on LBP outcomes [79] as implementation was only partially successful within one-year follow-up. Future programmes should incorporate team-scale multiprofessional training and organizational support to encourage knowledge transfer and sustainability.

Assessing the credibility of qualitative studies is challenging, as the way in which they are conducted and reported vary. This study complied with a guideline, combining deductive and inductive content analysis [48], and triangulating the findings with implementation theories. The collaboration within the research team enhanced its trustworthiness. Theoretical saturation was adequately reached during the content analysis, as the data exhibited repetition and the abstraction process could be conducted properly in line with the Elo and Kyngäs approach of combining categories, which further strengthened the study’s trustworthiness. The large representative sample of 51 participants across 12 focus groups ensured diverse perspectives and analytical depth [80–82]. However, we acknowledge that the variations in group size and duration are limitations. In addition, isolating the effects of multifaceted implementation strategies from contextual factors remains challenging.

The findings of the study are transferable to Finnish OHS and similar international contexts but may not be applicable to other cultural contexts. In Finland, 90% of employees are covered by OHS [83] and all employers are mandated to provide OHS for their employees. Healthcare systems differ across countries, which influences the factors that affect the implementation of BPS management. The HCPs’ participation in the intervention may have introduced response bias, as they might have emphasized positive changes or aligned their responses with study expectations. The interviews could not fully assess whether treatments were implemented as stated or intended, nor could they identify gaps in skills. In addition, power imbalances in OHS teams, particularly in dual roles (e.g. supervisor-staff) should be considered when interpreting the results [84]. We considered individual interviews, but previous studies have already primarily explored physiotherapists’ perceptions and focus group studies on multidisciplinary teams in OHS remain rare.

This study highlights the importance of helping HCP transition from a biomedical to a holistic BPS approach in OHS multidisciplinary teams. Knowing the facilitators of and barriers to this process can guide tailored implementation strategies. Future studies should (1) identify the factors that most significantly influence HCPs’ behaviour changes related to BPS management (2), develop strategies that reduce barriers and improve healthcare delivery at the organizational level, and (3) objectively evaluate the quality of patient assessment and the delivery of BPS management.

## Conclusion

One year after BPS management was initially implemented, the identified facilitators were primarily in the capability (e.g. skills, confidence to make treatment decisions) and motivation (e.g. communication, renewing professional identity) domains, and the barriers were predominantly in the opportunity domain, mainly due to the lack of team agreement and organizational and system-level constraints. To ensure that BPS management is effective and continues in OHS, future implementation strategies should integrate BPS practices into routine workflows. Support could be sustained through policy initiatives and reinforced through multidisciplinary collaboration. The results contribute to the development of targeted and multifaceted strategies to support the integration of BPS management into practice and to promote evidence-based care in OHS.

## Supplementary Information

Supplementary Material 1

Supplementary Material 2

Supplementary Material 3

## Data Availability

The anonymized data that support the findings of this study is provided within the manuscript and supplementary information files. Original transcipts are not openly available due to reasons of sensitivity.

## Abbreviations

BPS: biopsychosocial
COM-B: Capability, Opportunity, Motivation, Behaviour model
COREQ: The consolidated criteria for reporting qualitative research
HCP: Healthcare professional
LBP: Low back pain
OHP: Occupational health physician
OHS: Occupational health services
OPT: Occupational health physiotherapist
TDF: Theoretical Domains Framework
