# Supplementary material for: Capabilities, opportunities and motivations in implementing guideline-oriented biopsychosocial low back pain management: perceptions of occupational healthcare professionals after an educational intervention
Source: BMC Health Serv Res. 2025 Aug 29;25:1153. doi: 10.1186/s12913-025-13267-7 (PMC12398078; doi:10.1186/s12913-025-13267-7)
Supplement: Supplementary file 1 — Supplementary Material 1 [file 12913_2025_13267_MOESM1_ESM.docx]

**Supplementary Table 1a: Capabilities, opportunities, and motivations for HCPs and patients to form a common individual BPS understanding of LBP**

| **Target behaviour A. “HCPs and patients form a common individual biopsychosocial (BPS) understanding of low back pain (LBP)”** | | | |
| --- | --- | --- | --- |
| MAIN CATEGORY  COM-B model | GENERIC CATEGORY | SUBCATEGORY | OPEN CODES  (+) = facilitator  (-) = barrier  (0) = neutral |
| CAPABILITY  Including TDF categories: ‘Knowledge’, ‘Skills’ and ‘Behavioural regulation’* | **Ability to perform BPS clinical actions** | Awareness of multidimensional nature of LBP | Understanding of the multidimensional nature of pain (+) |
|  |  |  | Increased knowledge of imaging not necessary in non-specific LPB (+) |
|  |  |  | Awareness of patient-perspective related to the fear of pain and recovery (+) |
|  |  |  | Superficial knowledge of BPS approach in clinical practice (-) |
|  |  | Development of patient assessment, communication and interaction skills | Capability to rationalize the need for imaging (+) |
|  |  |  | Learning to assess patients with acute and persistent musculoskeletal pain (+) |
|  |  |  | Learning validation skills (+) |
|  |  |  | Considering patient’s life situation, workability and functional ability in the assessment (+) |
|  |  |  | Recognizing patient’s fear avoidance behaviour (+) |
|  |  |  | Developing courage to ask novel questions (+) |
|  |  |  | Developing courage to encourage the patient to trust in the back (+) |
|  |  |  | Not having the skills to manage patient’s BPS issues (-) |
|  | **Self-reflection to build routines** | Reflection on past and new clinical behaviours | Criticality towards previous thoughts (+) |
|  |  |  | Identification of what skills had been missing (+) |
|  |  |  | Professional not anymore afraid of pain (+) |
|  |  |  | Learning how to normalize the pain (+) |
|  |  |  | Recognizing the therapeutic value of encouraging staying active and involved in work (+) |
|  |  |  | Developing capability to create hope in the patient (+) |
|  |  |  | Not holding back when difficult issues arise and allowing patients’ emotions to arise (+) |
|  |  |  | Recognizing old habits of invalidating speech (+) |
|  |  | Establishing sustainable clinical routines | Intention to use BPS approach within the given resources (time, number of appointments) (+) |
|  |  |  | Reorganizing ways of working (+) |
|  |  |  | Clear process of HCPs’ own way how to use BPS approach with patients (+) |
|  |  |  | Demands time to process new knowledge (-) |
|  |  |  | Demands time to become a natural part of the normal way of working (-) |
|  |  |  | Making the effort to pay attention to interaction (+) |
|  |  |  | Challenging oneself to try new ways of working (+) |
|  |  |  | Continuously practising to work with new focus (+) |
| OPPORTUNITY  Including TDF categories: ‘Environmental context and resources’ and ‘Social influences’ | **Organizational resources to provide and monitor care** | Time constraints limiting comprehensive BPS evaluation | Insufficient time resource for an individual, multidimensional BPS assessment (-) |
|  |  |  | Insufficient time resource to develop solutions together with the patient (-) |
|  |  | Organizational level monitoring of OHS resource use (specialized care, imaging, and costs) | Organizational directive to book 30 min for OHPs’ first appointment for patients with LBP (+) |
|  |  |  | Organizational level monitoring of the number of referrals to OPT (+) |
|  |  |  | Organizational level monitoring of the sick leaves and care-related costs (+) |
|  |  |  | Monitoring the content of specialized care and imaging (+) |
|  |  |  | Easy access for patients to imaging (-) |
|  | **Supporting social context** | Patient’s responses influencing professional behaviours and decision-making | Understanding of lifestyle stressors and relaxation making sense for patient (+) |
|  |  |  | Contradictory instructions and explanations from other HCPs (-) |
|  |  |  | Positive patient feedback on the multidimensional pain explanation (+) |
|  |  |  | The patient’s negative expectations (wanting to retire and passive therapies) (-) |
|  |  | Support from professional networks | No support from other professions to BPS explanation for pain (-) |
|  |  |  | Authorities and opinion leaders supporting the BPS understanding of LBP (+) |
|  |  |  | HCP follow evidence and media (+) |
|  | **Educational materials modifying thoughts and beliefs** | Patient education booklet reducing fear and reliance on imaging | Experience that the use of the patient education booklet reduces patient’s fears (+) |
|  |  |  | Experiencing the patient education booklet useful (+) |
|  |  |  | Experiencing the patient education booklet important for OPT’s plausibility considering the discussion about the need of imaging (+) |
|  |  |  | Increases OPTs threshold to refer the patient to OHP for imaging purposes (+) |
|  |  | Visual educational tools enhancing patient understanding and self-awareness | Experiencing use of educational resources such as “Explain Pain” videos effective and well-received by patients (+) |
|  |  |  | Using video recordings during OPT sessions to help patients by revealing the difference between thoughts and behaviours leading to common understanding (+) |
| MOTIVATION  Including TDF categories: ‘Social/professional role, identity’, ‘Beliefs about capabilities’, ‘Intentions’, ‘Goals’, ‘Beliefs about consequences’ and ‘Emotions’** | **Renewing professional identity** | Expanding professional role, confidence and boundaries | Role of the OPT developed from bio to biopsychosocial (+) |
|  |  |  | Role of the OPT developed from lecturing to person-centred (+) |
|  |  |  | Expanding confidence and boundaries (+) |
|  |  |  | OHPs start giving BPS patient education (+) |
|  |  | Remaining in familiar professional role and within boundaries | Familiar professional role (0) |
|  |  |  | Passive professional role: OHPs role is to follow progress of rehabilitation (-) |
|  |  | Developing professional identity | Changing approach to encountering all patients (+) |
|  |  |  | HCPs experiences benefit of utilizing validation skills in their personal life (+) |
|  | **Person-centred communication and partnership** | Person-centred communication improves collaboration, treatment results and patient satisfaction | Creating a good therapeutic relationship at the beginning helps the collaboration in the future (+) |
|  |  |  | Patient-satisfaction increases through responding to worries (+) |
|  |  |  | Recognizing patient’s pain behaviours leading to conversations with patients (+) |
|  |  |  | When patient’s understanding is considered surprising facilitating key factors to successful rehabilitation are identified (+) |
|  |  | Rising interest in patient perspectives and engagement | Valuing individual patient encounter (+) |
|  |  |  | Rising interest in patient’s understanding (+) |
|  |  |  | Interest in patient’s life situation and goals (+) |
|  | **Aligned individual and organizational goals** | Establishing quality interaction goals in patient encounters | Goal of having a shared understanding with patient (+) |
|  |  |  | Paying attention to the nature of messages (+) |
|  |  |  | Putting an effort on giving positive feedback (+) |
|  |  |  | Raising precaution of words when writing in the patient records (+) |
|  |  | Organizational goals supporting clinical practices | Consistent fit of BPS model with the unified goal of the organization (+) |
|  |  |  | Setting organizational goals related to use of BPS approach (+) |
|  |  |  | Evaluating benefits and challenges of BPS approach in OPT (+) |
|  | **Reflecting on emotions** | Emotional responses to increased understanding (concern, anxiety) | Worry of the patients’ fear-avoidance behaviour (0) |
|  |  |  | Anxiety when patient misunderstands HCP’s words (0) |
|  |  | Professional satisfaction, sense of adequacy and calmness | Having a sense of peace (+) |
|  |  |  | Feeling of doing the job well (+) |
|  |  |  | Feeling of adequacy as a professional (+) |
|  |  |  | Natural and easy to encounter patients (+) |
| COM-B=Capabilities, Opportunities, Motivations, Behaviour -model; BPS=biopsychosocial; LBP=low back pain; HCP=healthcare professional; OHP=occupational health physician; OPT=occupational health physiotherapist; TDF=Theoretical Domains Framework  *TDF domains not identified in the analysis process: ‘Memory, attention and decision processes’  **TDF domains not identified in the analysis process: ‘Optimism’ and ‘Reinforcement’ | | | |

**Supplementary Table 1b. Capabilities, opportunities, and motivations for the systematic use of risk stratification tools at an early stage in the assessment of patients with LBP**

| **Target behaviour B. “HCPs use of risk stratification tools systematically at an early stage in the assessment of patients with LBP”** | | | |
| --- | --- | --- | --- |
| MAIN CATEGORY  COM-B model of behaviour | GENERIC CATEGORY | SUBCATEGORY | OPEN CODES  (+) = facilitator  (-) = barrier  (0) = neutral |
| CAPABILITY  Including TDF categories: ‘Knowledge’, ‘Skills’, ‘Behavioural regulation’ and ‘Memory, attention and decision processes’ | **Capacity to apply stratification tools in addressing risk factors** | Knowledge and ability to use the tools | Understanding the meaning of the tools (+) |
|  |  |  | Non-awareness of the tools (-) |
|  |  |  | Nonfamiliar with the stratified model of care (-) |
|  |  | Using tools to clarify LBP complexity and workability | Gives a lens to something that is difficult to describe in words (+) |
|  |  |  | Important factors related to pain problem and workability can be discussed with their proper names (+) |
|  | **Adaptations in risk stratification tool use** | Conscious decision to apply the tools systematically | Systematically using the tools in workability assessment (+) |
|  |  |  | Using the tools as a homework for the next appointment (+) |
|  |  | Conscious decision to apply the tools to selected patients | Using the tools with new patients (0) |
|  |  |  | Using the tools with patients with persistent LBP (0) |
|  |  |  | Choosing from a variety of available questionnaires (0) |
|  |  | Conscious decision not to apply the tools at early stage | Experience of tools not always suitable for patients with acute pain (-) |
|  |  |  | Experience of need to build up trust before discussing about psychosocial issues (-) |
|  |  |  | The HCP’s preference to interview instead of using the tool (-) |
|  |  | Memory and decision making of using the tools in clinical routines | Assessment of patients' body control more important in the OPT’s work (-) |
|  |  |  | The evaluation of medication and sick leave more important in the OHP’s work (-) |
|  |  |  | HCPs experience the tools too complicated to use (-) |
|  |  |  | Not remembering how to score the tools (-) |
|  |  |  | Forgetting to use the tools (-) |
|  |  |  | No time to systematically use the tools and mark the scores to patient records (-) |
|  |  |  | Clear process of HCPs’ of using the tools and marking the scores to patient records (+) |
| OPPORTUNITY  Including TDF categories: ‘Environmental context and resources’ and ‘Social influences’. | **Organizational or team agreement on risk stratification tool use** | Team agreement how and when apply tools | Agreement of the work distribution among professionals of tool use (+) |
|  |  |  | No team level agreement (-) |
|  |  | Organizational policies guiding tool use | Tools included in the organization’s electronic questionnaires (+) |
|  |  |  | No organization level agreement how to use the tools (-) |
|  |  |  | No possibilities to influence on organizational level decisions considering policies to use the tools (-) |
|  |  |  | No policy on marking the scores to electronic patient records (-) |
|  | **Supporting social context** | Patient responses influencing professional behaviours and decision-making | Patient does not find the questions of the tool relevant (-) |
|  |  |  | Patient questions the need of the tool (-) |
|  |  | Social and system-level influences encouraging tool use | PRM specialists require the use of the tool before consultation (+) |
|  |  |  | Insurance companies prefer the use of measurements for assessment of work disability (+) |
| MOTIVATION  Including TDF categories: ‘Social/professional role, identity’, ‘Intentions’, ‘Goals’ and ‘Beliefs about consequences’* | **Using risk stratification tools to facilitate the rehabilitation process** | The tools serving as conversation starters | Aids conversation (+) |
|  |  |  | Allows to ask about difficult issues because the patient has produced the information (+) |
|  |  | The tools aid in clinical reasoning | Awakens to consider the patient’s situation as a whole (+) |
|  |  |  | Helpful in clinical reasoning (+) |
|  |  | The systematic use of the tools improves treatment planning, quality of work and saves time | Ensures the treatment line is considered in advance (+) |
|  |  |  | Enables making action plan at early stage (+) |
|  |  |  | Systematic mapping of psychosocial factors improves quality of work (+) |
|  | **Aligned individual and organizational goals** | Establishing goals for systematic use of the tools | Individual goal to systematically use the tool for risk stratification (+) |
|  |  |  | Opportunity to guide the direction and extensiveness of the rehabilitation process (+) |
|  |  | Organizational goals supporting clinical practices | Goal to apply systematically in the organization through electronic questionnaires (+) |
|  |  |  | Training HCPs nationwide to facilitate the systematic use of tools in organization (+) |
| COM-B=Capabilities, Opportunities, Motivations, Behaviour -model; TDF=Theoretical Domains Framework; BPS=biopsychosocial; LBP=low back pain; HCP=healthcare professional; OHP=occupational health physician; OPT=occupational health physiotherapist; PRM=physical and rehabilitation medicine specialist  *TDF domains not identified in the analysis process: ‘Beliefs about capabilities’, ‘Optimism’, ‘Reinforcement’ and ‘Emotions’ | | | |

**Supplementary Table 1c. Capabilities, opportunities, and motivations for multidisciplinary collaboration targeting an individualized plan for patients with LBP**

| **Target behaviour C. “Multidisciplinary collaboration targeting an individualized plan for patients with LBP”** | | | |
| --- | --- | --- | --- |
| MAIN CATEGORY  COM-B model of behaviour | GENERIC CATEGORY | SUBCATEGORY | OPEN CODES  (+) = facilitator  (-) = barrier |
| CAPABILITY  Including TDF categories:  ‘Knowledge’, ‘Skills’, ‘Memory, attention and decision processes’* | **Confidence in managing treatment decisions** | Therapy skills aligned with patient goals | Targeting therapy to patient’s everyday functioning (+) |
|  |  |  | Patient’s individual goals involved in the treatment plan (+) |
|  |  | Confidence in taking responsibility for the treatment process | OHP’s courage to take responsibility of the treatment line (+) |
|  |  |  | Trust in multidisciplinary team capabilities before referring the patient to imaging (+) |
|  |  |  | Patience to see the results of the rehabilitation before referring to specialized care (+) |
|  |  |  | OPT’s confidence of being capable of treating more challenging patients (+) |
|  |  | Decision-making and prioritization of OHS resources | OHP’s decision to use all needed multidisciplinary resources in the unit (+) |
|  |  |  | OPTs not part of decision process of referring the patient to specialized care or use of multidisciplinary resources (-) |
|  |  |  | The OPT experience of being an outsider of the OHS team (-) |
| OPPORTUNITY  Including TDF categories: ‘Environmental context and resources’ and ‘Social influences’ | **System-level drivers** | System level structural barriers for rehabilitation in OHS | The system does not sufficiently support early-stage rehabilitation in OHS (-) |
|  |  |  | System level policy not allowing more than three OPTs’ appointments in Finnish OHS (-) |
|  |  | Variability in practices based on client company size | Handling small client companies in the unit/team (patient follow-up difficult to organize due to costs, no regular team meetings) (-) |
|  |  |  | Large companies (rigid, not possible to create practices locally) (-) |
|  | **Organizational resources to provide and monitor care** | Physiotherapy and rehabilitation resources within the unit | Direct access to physiotherapy without delays (+) |
|  |  |  | Long wait for patients to OPT (-) |
|  |  |  | Flexibility to use OPT according to needs (+) |
|  |  |  | Insufficient resources in the unit for BPS trained OPTs (-) |
|  |  | Collaboration outside OHS to ensure active rehabilitation | Collaboration with BPS trained HCPs outside OHS (+) |
|  |  |  | HCP cannot influence on the content of rehabilitation outside OHS (active/passive) (-) |
|  | **Individualized treatment planning for high-risk patients** | Access to new tools for individualized treatment planning with patient and a multidisciplinary team | Using risk stratification in multidisciplinary treatment planning (+) |
|  |  |  | OPT using Patient-Specific Functional Scale for identification of functional goals together with the patient (+) |
|  |  |  | Importance of including goals for leisure time, not only work-related functions (+) |
|  |  |  | Making the treatment plan based on risk stratification in collaboration with all team members (+) |
|  |  | Influence of authorities in treatment planning | OHP with authority helping to convince the patient for the treatment plan making it easy for OPT to proceed with rehabilitation (+) |
|  |  |  | Orthopaedic surgeon consultations provide non-individualized treatment plans (-) |
|  |  | Creating continuity of care through organized follow-ups | OPT creating trust by giving direct phone number for patient (+) |
|  |  |  | Organizational policy to contact the patient after the OPT appointment by phone (+) |
|  |  | Systematic monitoring of treatment processes and case management of high-risk patients | Systematic company level monitoring of sickness absence rates and patients with M-diagnosis are contacted by OPT (+) |
|  |  |  | No organizational agreement of care pathway or active monitoring of high-risk patients (-) |
|  |  |  | OHN / OHP responsible for the care pathway for high-risk patients (+) |
|  |  |  | No case manager for high-risk patients (-) |
|  |  | Available care pathways and options for high-risk patients in the unit | Clear process of stratified care agreed in the organization (+) |
|  |  |  | Non-existing or unclear rehabilitation processes (-) |
|  |  |  | Psychologists not actively part of treatment of patients with LBP (-) |
|  |  |  | Starting BPS multidisciplinary group rehabilitation in the unit (+) |
|  |  |  | No multidisciplinary group activities in the unit (-) |
|  |  |  | Rapid multidisciplinary collaboration for high-risk patients (+) |
|  | **Stability in multidisciplinary collaboration** | Organizational and team stability ensuring continuity in multidisciplinary work | Regular interprofessional meetings ensuring continuity of care (+) |
|  |  |  | Multidisciplinary collaboration based on knowledge-transfer through electronic patient records (-) |
|  |  |  | Patients are treated by many different HCPs with high turn-over and short work periods (-) |
|  |  |  | No time or possibilities for multidisciplinary meetings due to financial reasons (-) |
|  |  |  | Changes in job descriptions (-) |
|  |  |  | Flood of information (digital leap) (-) |
|  |  |  | Reforms in the organizations (-) |
|  |  | Interprofessional identity and shared knowledge of BPS management | Multidisciplinary BPS training involving both OPTs and OHPs from the same unit/team (+) |
|  |  |  | Interprofessional group identity and peer support of ‘clinical champions’ (+) |
|  |  |  | Disconnection between BPS training and routine OHS teamwork (-) |
|  |  |  | Sharing knowledge of BPS approach with interprofessional meetings (+) |
|  |  |  | Influential HCPs facilitating the interprofessional knowledge transfer (+) |
|  |  |  | Continuing BPS education in the organization to unify and harmonize the treatment pathways and multidisciplinary collaboration (+) |
|  |  |  | Challenge of leading others in BPS approach (-) |
|  |  |  | Challenges in sharing of knowledge and experiences within the large unit (-) |
| MOTIVATION  Including TDF categories: ‘Social/professional role, identity’, ‘Beliefs about capabilities’, ‘Intentions’, ‘Goals’ and ‘Beliefs about consequences’  ** | **Renewing professional identity** | Expanding role, confidence and boundaries | The role of OPT more versatile, not focused only to ergonomics (+) |
|  |  |  | OHP’s experience that the role of OPT is emphasized in addressing pain behaviour, fears, and treatment of LBP (+) |
|  |  |  | OPT confidence to bring out the expertise more broadly in multidisciplinary collaborative situations (+) |
|  |  |  | Positive experiences of OPT applying BPS approach with patients with both mental and musculoskeletal issues (+) |
|  |  |  | Psychologist not always needed when OPT use BPS approach (+) |
|  | **Applying BPS management to enhance use of physiotherapy and multidisciplinary collaboration** | Increasing physiotherapy use and improving patient outcomes and return-to-work | Increases use of physiotherapy (+) |
|  |  |  | Accelerates patients’ return to work (+) |
|  |  |  | Decreases number of patients waiting for specialized care (+) |
|  |  | Enhancing multidisciplinary collaboration | Multidisciplinary collaboration more effective than before (+) |
|  |  |  | Increases referrals between HCPs (OHN-OPT-OHP-psychologist) (+) |
|  |  |  | Increases OHN confidence in referring LBP patients more quickly to an OPT instead of OHP (+) |
|  | **Encountering expectations and attitudes towards LBP management** | Expectations regarding LBP treatment | Raising positive patient expectations towards multidisciplinary BPS management of LBP (+) |
|  |  |  | Avoiding early imaging due to low benefit and potential harms related to imaging (+) |
|  |  |  | Referring to imaging to support patient’s commitment to the treatment process (-) |
|  |  |  | OPTs experience patients prefer advice and active rehabilitation (+) |
|  |  |  | OPT experience patients to expect passive treatments even he/she prefers self-care and active exercise (-) |
|  |  | Encountering HCP attitudes towards BPS management | Enthusiasm of having something new for the patients (+) |
|  |  |  | Increasing interest of team members after successful patient experiences (+) |
|  |  |  | Buy-in the goal of finding patients’ abilities and opportunities despite the LBP (+) |
|  |  |  | Hunger for information of BPS approach (+) |
|  |  |  | Low enthusiasm and disbelief among multidisciplinary teams to BPS approach (-) |
|  |  |  | Shock, frustration and anger for not being able to use the skills from previous trainings (-) |
|  |  |  | Waiting for the research results from OHS context (-) |
| COM-B=Capabilities, Opportunities, Motivations, Behaviour -model; TDF=Theoretical Domains Framework; BPS=biopsychosocial; LBP=low back pain; HCP=healthcare professional; OHS=Occupational health services; OHP=occupational health physician; OPT=occupational health physiotherapist; OHN=occupational health nurse  *TDF domains not identified in the analysis process: ‘Behavioural regulation’  **TDF domains not identified in the analysis process: ‘Optimism’, ‘Reinforcement’ and ‘Emotions’ | | | |
