# Supplementary material for: Capabilities, opportunities and motivations in implementing guideline-oriented biopsychosocial low back pain management: perceptions of occupational healthcare professionals after an educational intervention
Source: BMC Health Serv Res. 2025 Aug 29;25:1153. doi: 10.1186/s12913-025-13267-7 (PMC12398078; doi:10.1186/s12913-025-13267-7)
Supplement: Supplementary file 2 — Supplementary Material 2 [file 12913_2025_13267_MOESM2_ESM.docx]

| **Table 1a: Capabilities, opportunities, and motivations for HCPs and patients to form a common individual BPS understanding of LBP** | | |
| --- | --- | --- |
| SUBCATEGORY | OPEN CODES | Quotation translated in English |
|  | (+) = facilitator  (-) = barrier  (0) = neutral |  |
| Awareness of multidimensional nature of LBP | Understanding of the multidimensional nature of pain (+) | “Well, for me at least, it was very familiar and homey thinking or in line with my way of interacting with clients - to consider the client as a whole, to map out their life story, and to think more broadly about the biopsychosocial factors.” OPT9PC4Y |
|  | Increased knowledge of imaging not necessary in non-specific LBP (+) | "Well, I have at least been confirmed that it [imaging] is not of primary importance when it has been clinically established that there are no signs of danger." OPT12PUC2Y |
|  | Awareness of patient perspective related to the fear of pain and recovery (+) | "Somehow it's about the perspective that for the physician, the patient with back pain is a very ordinary patient, and we treat them like an ordinary patient and say certain things. Then, for the patient, even if it looks like an easy back pain case, [for the patient] it is actually a difficult and frightening situation. If you can somehow acknowledge and understand that during the consultation, the patient is more likely to recover better." OHP14PC2Y |
|  | Superficial knowledge of BPS approach in clinical practice (-) | OPT25PC4N: "...since I wasn't there [BPS education] and was only in that [unit visit] training, which was about 2 hours long, I at least was left wondering what this was all about. I personally needed a bit more information to be able to implement it as the goal of this study seemed to be. It didn't quite make sense to me at first, but then [colleague] gave me a summary of it, and after that, I finally understood what it was about... But I would still like more clarity, since this is a study, on how this framework is supposed to be applied." OPT23PC4Y: "Similarly, I feel that those who are doing it should have received the same type of training from the beginning, so that we would all be on the same page from the start." OPT25PC4N: "Yes." OPT23PC4Y: "Both physicians and OPTs." |
| Development of patient assessment, communication and interaction skills | Capability to rationalize the need for imaging (+) | "Yes, I also feel that after that training... indeed, there were more ways to explain to the clients why imaging might not be useful at this stage." OPT21PC2Y |
|  | Learning to assess patients with acute and persistent musculoskeletal pain (+) | "Although this study was about low back pain, this model is, of course, applicable to other musculoskeletal issues as well. Whether it is neck pain, shoulder pain, hip pain, or even toe pain, we approach the pain, patient interview, and encounter in the same way. It has indeed affected the treatment of all kinds of [musculoskeletal] conditions." OPT20PC2Y |
|  | Learning validation skills | "Well, maybe it's about using these validation phrases consciously... " OHP4PC2Y |

|  | Considering patient’s life situation, workability and functional ability in the assessment (+) | "Maybe I can say from my own experience that how it would have affected the practice is that, in addition to clinical examination, I might have invested more in the treatment and support of the patient. And I have also personally guided some relaxation exercises and similar practices during the consultation. Probably interviewed the patient better, not just focusing on the acute issue but also exploring their life situation or how the pain affects their functionality. That is also a core aspect in OHS, the impact on functionality. We have invested in that interview and tried to support the patient towards normal work capacity." OHP3PC1Y |
| --- | --- | --- |
|  | Recognizing patient’s fear avoidance behaviour (+) | "Yes, I feel that I have paid more attention to the fear of pain and avoidance behaviours than before. Somehow, I got that message from their [patient’s] speech, and it has perhaps increased the discussion about it as well." OHP10PUC2N |
|  | Developing courage to ask novel questions (+) | "Well, it has been very effective with these persistent pain patients and revolutionized how I have worked before, providing a lot of additional tools, especially for back issues... Knowing how to release those [tensions], understanding how to start releasing them, and knowing how to ask questions. The kind that I might not have known before, which give more information." OPT5PC3Y |
|  | Developing courage to encourage the patient to trust in the back (+) | "It has changed, changed to encouraging movement without tension, without fear. Caution. The word 'caution' has completely disappeared. I think that reflects the change the most. I no longer use the word 'caution'." OPT4PC2Y |
|  | Not having the skills to manage patient’s BPS issues (-) | "And then when a really difficult patient comes, who is struggling with their pain, I don't necessarily know how to handle those clients in this study. So, I have filled out the forms, but I have then referred such cases to [BPS trained physiotherapists], as they know how to handle them better. I am somewhat straightforward in such matters." OPT17PC2N |
| Reflection on past and new clinical behaviours | Criticality towards previous thoughts (+) | "Maybe these core muscle thinking patterns changed quite radically. Yes, I admit that at some point I also engaged in that kind of “core thinking”, like keeping the back straight while squatting. That has changed radically in my own guidance... each of these [LBP patients] has received instructions from their OHS professionals to be careful, keep the back straight, and not to bend the back when lifting. Tighten the abdominal muscles, and a couple of them had the comment that if I tighten my abdominal muscles, I can't breathe. And when we went through the breathing exercises, breathing along with the movements, it helped relax the body muscles. But it probably stems from the fact that, including myself, I previously gave such information to tighten your abdominal muscles strongly and maintain that. The neutral position of the back." OPT4PC2Y |
|  | Identification of what skills had been missing (+) | "It has definitely changed a lot and helped in dealing with clients, especially those who catastrophize. If I think about the past, I didn't recognize that they were catastrophizing, and this approach has helped me in how I interact with them. You can see that the situation calms down when you just interview and talk to the client about things that might be familiar to them but how they have understood them. This has helped me personally handle many situations much more easily than before, by listening and talking with the client. It's an extremely important tool that I actually use all the time. It is my approach in the interviews, to approach the client." OPT7PC4Y |
|  | Professional not anymore afraid of pain (+) | "To dare to be firm [as professional], to not be afraid that patients back will break." OPT22PC2Y |
|  | Learning how to normalize the pain (+) | "This model now brings understanding and aims to restore everyday functionality, starting with small daily movements and changes when living with persistent pain, or at least to normalize the experience of it." OHP14PC2Y |
|  | Recognizing the therapeutic value of encouraging staying active and involved in work (+) | “[Anonymized OPT] has been implementing this model in our unit. He has told us about this training, and… encouraging movement and avoiding being sedentary, such as staying on the couch, is something we should promote. This has definitely helped in our work and improved collaboration." OHN7PC2N  "We definitely have to consider the individual's needs and think about relevant coping strategies. I believe that for OPTs, it cannot be just clinical approach but rather about coping and encouragement as in this treatment approach. In many cases, work itself can be much more rehabilitative than, for example, sick leave." OPT17PC2N |
|  | Developing capability to create hope in the patient (+) | "Every person has certain dreams and wishes that they may have avoided or tried to avoid for years, and when those are verbalized, suddenly the person might have an 'aha' moment. It can be hopeful and encouraging to realize that it might be possible to achieve these physical dreams that they haven't dared to think about." OHP4PC2Y |
|  | Not holding back when difficult issues arise and allowing patients’ emotions to arise (+) | "Maybe with this method, there has been a lot more of people crying during consultations than before. When those difficult issues come up. Probably the fact that the encounters are better nowadays, and you focus more on letting things come up if they arise, because they are important." OPT20PC2Y |
|  | Recognizing old habits of invalidating speech (+) | “But what I have learned and am still learning is precisely how to talk to the client. I realized that it's easy to unintentionally invalidate them, so I've been trying to improve that and focus a lot on giving positive feedback. I have also avoided mentioning observations that could have a nocebo effect. So, in that way, it has had an impact.” OPT9PC4Y |
| Establishing sustainable clinical routines | Intention to use BPS approach within the given resources (time, number of appointments) (+) | OPT12PUC2Y: Much more time can be spent with the client without having to watch the clock and worry about what functional tests we still have time to do. But that doesn't matter because then there is the possibility to schedule another visit and continue from there. The important phase is making that contact, allowing time to open up and discuss things calmly, and to go through the client's story and narrative. OPT6PUC2Y: And then you don't need to the next time. OPT12PUC2Y: Right. OPT10PUC2Y: Often, when the client starts to comprehensively understand their own situation, the discussion can reveal things that, by the next visit, even basic movements can show significant improvement. |
|  | Reorganizing ways of working (+) | "It changed my time management, particularly in the examination situation, the consultation. It's precisely about the dialogue and mapping, listening to the person's wishes and needs. It's not always just about the joint movement that determines work capacity; there's so much more involved." OPT4PC2Y |
|  | Clear process of HCPs’ own way how to use BPS approach with patients (+) | "Well, the first visit is that we usually talk for half an hour, even forty minutes, to discuss. We identify the problem area, address it immediately. On the next visit, if everything has gone well, it might be two weeks, three weeks, or it could be a week, depending on the psychosocial factors. If they feel it's beneficial, we move forward. We increase the resistance, which means progressing, creating more challenges. The last visit could be, with maybe a month in between if everything goes well. We can bring the appointment forward or if it's very difficult case, it could be every other week." OPT5PC3Y |
|  | Demands time to process new knowledge (-) | "I think it takes time for us to adopt it more and to spread it more widely here." OPT19PC2Y |
|  | Demands time to become a natural part of the normal way of working (-) | "...for me personally, it has required time to become natural and something that I have integrated it into my normal practice." OPT19PC2Y |
|  | Making the effort to pay attention to interaction (+) | "What I have learned and am practicing is precisely how I talk to the customer, as it is very easy to invalidate them. I have tried to improve that and focus a lot on giving positive feedback, leaving out observations that might have nocebo effects." OPT9PC4Y |
|  | Challenging oneself to try new ways of working (+) | "Well, it is not really difficult to implement, but learning to dare to ask and challenge oneself, that is difficult. To dare to let go of what you have previously thought and to start trying those things boldly. Especially with more difficult issues, to dare to challenge." OPT5PC3Y |
|  | Continuously practising to work with new focus (+) | "Yes, and you always have to revisit the materials, having those 'aha' moments that deepen the understanding. You never truly finish, and we can't just float here. It seems like you have to delve deeper, with the abundance of material now provided to us." OPT12PUC2Y |
| Time constraints limiting comprehensive BPS evaluation | Insufficient time resource for an individual, multidimensional BPS assessment (-) | “Likewise, in OHP' appointments, the 20 minutes are not enough to thoroughly interview and listen; the time just runs out.” OHP13PC2Y |
|  | Insufficient time resource to develop solutions together with the patient (-) | OHP12PC2N: "Well, it's exactly about the time, many times you would like to start talking about deeper issues but you know where it leads (laughs)." OHP13PC2Y: "Yes, sometimes it's really like you don't want to stir things up." OHP12PC2N: "No." OHP13PC2Y: "It's just that you know we don't have the time. Sometimes, if I clearly feel that this person is not well, we do have the option to arrange another appointment and schedule a longer time for it." |
| Organizational level monitoring of the use of resources (specialized care, imaging, and costs) | Organizational directive to book 30 min for physicians’ first appointment for patients with LBP (+) | "More time has been allocated for patients with LBP. The organizational directive has been that the physician's appointment should be 30-40 minutes." OHP3PC1Y |
|  | Organizational level monitoring of the number of referrals to OPT (+) | "And this model has been in use for years in our organization, where the OHP refers all patients with LBP to the OPT for guidance and advice. We monitor the amount of these referrals and how well this has been implemented. In practice, it has been increasingly implemented year after year." OHP4PC2Y |
|  | Organizational level monitoring of the sick leaves and care-related costs (+) | "Similarly, we monitor the sick leaves of these companies monthly, every three months, and all [patients in ICD-10] M-group are then referred to OPTs. Occupational health contacts them to see if the treatment has started." OHP4PC2Y  "I think it has, and I believe it also influences our OHS agreements where we have to consider the costs. That is probably another reason why imaging has decreased." OPT22PC2Y |
|  | Monitoring the content of specialized care and imaging (+) | "The only thing is that if they get to see a specialist, then imaging is almost automatic [laughs]. Of course, I follow some of them because I get consulted, so I follow some of the patient records." OHP3PC1Y |
|  | Easy access for patients to imaging (-) | "I would say that I haven't noticed that our practices have changed regarding this, but more and more people are going for those imaging investigations. They want to have it and they pay for themselves." OHP12PC2N |
| Patient education booklet reducing fear and reliance on imaging | Experience that the use of the patient education booklet reduces patient’s fears (+) | "This study involves this patient education booklet... Some LBP patients gave good feedback about it. In the consultation, we discuss various things, and it summarizes many points very well. When they read it at home, it gives them courage and a positive expectation for the future. Fears are reduced as well. It is good material. Such encouraging material is always needed. Probably, we would hope that such a booklet will be available in the future." OHP19PC2Y |
|  | Experiencing the patient education booklet useful (+) | "Yes, it is almost like a patient education booklet that could be given to everyone, it could almost be more of a rule than an exception, because it provides a way of thinking and understanding about how common back pain is, that I am not alone and this is the way to treat it, and there is no need to fear it. So, it could almost be more of a rule than an exception." OPT17PC2N |
|  | Experiencing the patient education booklet important for OPT’s plausibility considering the discussion about the need of imaging (+) | "Just to avoid unnecessary imaging. The [patient education booklet] was probably good for the client. The same things had been discussed with the client, so it was probably good for them to see that it wasn't just me saying it." OPT22PC2Y |
|  | Increases OPTs threshold to refer the patient to OHP for imaging purposes (+) | “Kind of self-confidence that I’m able to say, 'you don't need imaging', the threshold has risen really, really high for to recommend for patient that you go back to the OHP now, let's get it imaged first, before starting the rehabilitation. It's really high these days." OPT20PC2Y |
| Visual educational tools enhancing patient understanding and self-awareness | Experiencing use of educational resources such as "Explain Pain" videos effective and well-received by patients (+) | "I've also used things like Explain Pain, and I've sent clients links to those, um, websites and some YouTube videos that have been translated... I've shared those links with clients as a sort of homework for them to familiarize themselves with. The feedback from clients has been quite positive.” OPT20PC2Y |
|  | Using video recordings during OPT appointments to help patients by revealing the difference between thoughts and behaviours leading to common understanding (+) | "And I've often used video recordings more, where I show the client during the session what they are doing. It often leads to an 'aha' moment for the client... In my experience, patients respond well to this, and it leads to insights. I've received more positive feedback about this approach, and sometimes they even look at old photos or ask someone to record them at home or work, where the symptoms occur. It's a realization when they see it. They imagine they're moving or using their body in a certain way, and then they see what they're actually doing." OPT11PUC2Y |
| Patient’s responses influencing professional behaviours and decision-making | Understanding of lifestyle stressors and relaxation making sense for patient (+) | "Yes, the physiology of persistent pain has opened up a bit differently, and then if you discuss it and the customer talks about all the things that are affecting it, it somehow gives them a more peaceful feeling. And then when you see that the customer calms down and understands." OPT12PUC2Y |
|  | Contradictory instructions and explanations from other HCPs (-) | OPT23PC4Y: "And indeed, it's quite often, you know, that we encounter these people who say, 'The physician said I shouldn't do this,' or 'The physician said I shouldn't do that,' or 'The physician says my back is really bad.' These cases still come up." OPT25PC4N: "Maybe more so with physicians than with PTs. I see it that way, that our profession is generally like that." OPT9PC4Y: "Yes, and we get these instructions asking us to recommend exercises where the back shouldn't move this way or that way." OPT25PC4N: "Yes, yes." OPT9PC4Y: "And the client has heard them and asks, 'The physician said not to move this way or that way,' so it's a really awkward situation." OPT25PC4N: "Yes." OPT9PC4Y: "To say that probably what was meant is something like..." OPT23PC4Y: "As if to defend it." OPT25PC4N: "Now you can't contradict what the physician just said." OPT9PC4Y: "And just as I still had to stick to my point that we shouldn't avoid those movements, it was such a weird situation that completely opposite things are being said." OPT25PC4N: "Yes, indeed." OPT23PC4Y: "There are these conflicting messages." |
|  | Positive patient feedback on the multidimensional pain explanation (+) | "Well, somehow their sense of self-efficacy definitely improves and, in general, they feel better about themselves. It opens the client's eyes to what else they might be able to do. It lowers the threshold to expand their activities and so on. And understanding that the pain and problems are not just mechanical and physical [laughs]. It also makes it easier for the clients, like 'that explains it' and 'it's nice to hear' – comments like that come up when we talk about these things." OPT9PC4Y |
|  | The patient’s negative expectations (wanting to retire and passive therapies) (-) | "OPT5PC3Y: There are also people who, no matter how hard you try, don't listen or see anything... OHP5PC3N: It's probably an inherent trait in some people that they are not really in touch with their deeper thoughts. They just want one-dimensional approach and get a massage and be done with it. OPT5PC3Y: Or they've given up. OHP5PC3N: Mmm. Then there are those who may have already built this idea in their minds that they want to leave working life by any means necessary. And then they have built a wall around that idea." |
| Support from professional networks | No support from other professions to BPS explanation for pain (-) | “And probably in cases of long-term LBP, you see that the need for care extends beyond OHS, especially if there have been symptoms that reduce functionality and workability for a long time. We then utilize external expertise, such as PRM specialists and physiotherapists from outside OHS. There, you notice a bit of confusion in clients about how to proceed, especially when one [professional] might say 'definitely not' while we have said something different here. You really have to think about your own words and how to continue. This is something we encounter repeatedly... Probably related to the use of the back specifically. If we instruct to return to normal way of using back as soon as possible." OHP1PC1Y |
|  | Authorities and opinion leaders supporting the BPS understanding of LBP (+) | It was open to everyone—OHNs, OHPs, and OPTs. There weren't many people there, so it was like that. But [anonymized name] managed to positively spread the message forward; I think it was a really good lecture. And then, next time, we had a national Skype training for our physiotherapists, which [anonymized name] held. And then there was [anonymized name]'s pain education, which... was somewhat linked to [anonymized name]'s talk. But those are what we have, and what I have been spreading here, and what [anonymized clinical champions] have been spreading in their unit." OPT4PC2Y |
|  | HCP follow evidence and media (+) | OHP12PC2N: “So, this new way of thinking has become quite familiar; I've also read about it elsewhere. OHP13PC2Y: Similar approaches are used for many functional disorders. OHP12PC2N: Yes. OHP13PC2Y: Whether it's fibromyalgia or something else, it can be used for conditions other than just back pain. OHP12PC2N: Yes. But I see it as a positive that we now have the opportunity to try it out.” |
| Expanding professional role, confidence and boundaries | Role of the OPT developed from bio to biopsychosocial (+) | "This has somehow broadened my awareness and I no longer pay attention to only to mechanics, instead I consider functional movements and whether there is that pain related fear, and what causes it." OPT9PC4Y |
|  | Role of the OPT developed from lecturing to person-centred (+) | “I invest quite a lot in such dialogical discussion, much more than before. Previously, it was more about advising and taking control of the situation, almost like lecturing the client. Now it’s more of a biopsychosocial approach with dialogue, which is good.” OPT4PC2Y |
|  | Expanding confidence and boundaries (+) | "And it encourages us as OPTs to be able to address issues that might fall more into the realm of psychology. In the past, I noticed that there was a hesitation to encroach on what was seen as someone else's area. But now, this has given me the confidence to say, hey, this is about a holistic approach to meeting the person and addressing their problems. I’ve started to emphasize this even more, or at least I’ve gained more courage in doing so. That's what I would say for myself." OPT25PC4N |
|  | Physicians starts giving BPS patient education (+) | "I give a certain amount of guidance in basic things to these people with acute back pain, who don’t seem to need the guidance of a PT at the beginning... acute pain phase self-care methods, relaxation techniques, and then walking, how to start, and gradually increase activity as the pain allows, those I always go through... And with these long-term clients who have had reduced work capacity for a long time and very severe symptoms with their back, it is really easy to get started with them. Since they are already somewhat strengthened, they know what needs to be done. So, that it will be fine. Maybe the awareness of acute pain management has improved." OHP1PC1Y |
| Remaining in familiar professional role and within boundaries | Familiar professional role (0) | “I haven't gained much from this. Because, of course, I’ve been a physician for such a long time.” OHP4PC2Y |
|  | Passive professional role: OHPs role is to follow progress of rehabilitation (-) | "And because we always refer the patient to physiotherapy when they are in the pain phase, we expect the professionals to provide the guidance, and then we monitor how the rehabilitation has progressed." OHP4PC2Y |
| Developing professional identity | Changing approach to encountering all patients (+) | “Yes, through training, I’d say that it has somewhat changed the entire patient interaction from my side, trying to understand the client's own thoughts and what the client themselves sees as causing their issues.” OHP1PC1Y |
|  | HCPs experiences benefit of utilizing validation skills in their personal life (+) | "I guess I listen more and then the treatment is more individualized. And it produces results. I’m still learning in my old age. This dialogue approach applies even in the company of friends." OPT4PC2Y |
| Person-centred communication improves collaboration, treatment results and patient satisfaction | Creating a good therapeutic relationship at the beginning helps the collaboration in the future (+) | "And I have noticed that when you create that relationship well at the beginning, even if things don't start off smoothly, it feels like you have more opportunities later on to change direction or try something different." OPT21PC2Y |
|  | Patient-satisfaction increases through responding to worries (+) | “It has indeed produced good results, the clients are very satisfied when they are listened to and their emotion is responded to.” OHP4PC2Y |
|  | Recognizing patient’s pain behaviours leading to conversations with patients (+) | "If I think about the past, I didn't recognize that they were catastrophizing, and this approach has helped me in how I interact with them. You can see that the situation calms down when you just interview and talk to the client about things that might be familiar to them, but how they have understood them." OPT7PC4Y |
|  | When patient’s understanding is considered surprising facilitating key factors to successful rehabilitation are identified (+) | "And indeed, by stopping to listen to the person and letting them contribute more content to the discussion, very surprising things emerge that are very significant for their rehabilitation in the end." OPT21PC2Y |
| Rising interest in patient’s perspective and understanding | Valuing individual patient encounter (+) | “Well, I have personally noticed that I pay much more attention to building the client relationship and, as [anonymized] mentioned, specifically focusing on the exercises [supporting that] and the return to activity in the context of the individual’s life. For example, through their hobbies, not directing the same exercises to everyone and not following the same path, but rather tailoring it specifically to the individual’s life, finding things that help them move forward through that." OPT21PC2Y |
|  | Rising interest in patient’s understanding (+) | "...the biggest thing that I have been thinking about is that the person always has a very significant anxiety about their back pain. Or some worry or thought that is constantly in the background and affects their actions, which we may not have previously paid so much attention to." OHP14PC2Y |
|  | Interest in patient’s life situation and goals (+) | “And then asking the person's own perceptions about what they think is behind the back pain. And then what they would like to do if there was no pain. This is also part of setting goals." OHP4PC2Y |
| Establishing quality interaction goals in patient encounters | Goal of having a shared understanding with patient (+) | OHP13PC2Y: "...which still requires learning on my part, trying to listen to the patient's own interpretation of what is causing this and what is related to it, and then being able to move forward from there." OPT19PC2Y: "And it is a big thing, their own understanding or vision, that this is what it is, and that's why it is. It defines their actions when they think what the issue is. If you can influence that immediately, their thought changes, and so does their behaviour, and consequently, the symptom as well." |
|  | Paying attention to the nature of messages (+) | "…not to create more catastrophizing in the situation and not to say that there is something wrong [in the spine] even if the imaging has been done... And maybe the message about the nature of the pain and the fear of pain, those related issues, has probably changed. And in a way, it also takes into account the whole other life alongside it." OHP13PC2Y |
|  | Putting an effort on giving positive feedback (+) | "Yes, the significance of wording is something that could still be considered even more actively. But I do think, or somehow, I have tried with each patient with LBP to find something specific that I can praise about their actions, presence, perseverance, or something similar. I think it changes the person’s whole presence when they realize, 'Oh, I can do this, and I am able to.'" OHP14PC2Y |
|  | Raising precaution of words when writing in the patient records (+) | OHP7PC4N: "What you mentioned is very important, that if there are good ideas or concerns arising in physiotherapy, they should be brought up because they are very useful and interesting. If it's something that can't be reasonably documented in the text, then the feedback should come through other means." OPT7PC4Y: "Mmm. Sometimes it's challenging to write that into the text because the texts are visible [for patients] through Kanta [electronic patient record system]. So, how do you document it?" |
| Organizational goals supporting clinical practices | Consistent fit of BPS model with the unified goal of the organization (+) | OPT23PC4Y: "As [organization], we have done quite a lot, not related to this project specifically, but focusing on the length of sick leaves." OPT25PC4N: "And these care pathways." OPT23PC4Y: "These care pathways have been built, but they are part of other projects, yet they have been ongoing within the organization all the time. I believe that some attention has also been given to this among the physicians." OPT24PC4N: “And then [as OHS organization], we've introduced models of substitute, lightened, and modified work to companies. So it's part of our everyday efforts to advance this with companies.” |
|  | Setting organizational goals related to use of BPS approach (+) | "I've been discussing with [anonymized head of the unit], and we both feel like winning the lottery by being part of this research project and getting this information. We always strive to do things better, which applies to looking at ourselves in the mirror, making time to discuss these cases extensively, and developing this together. It feels like we are heading in the right direction." OPT11PUC2Y |
|  | Evaluating benefits and challenges of BPS approach in OPT (+) | "We did group work where we discussed the benefits of this BPS approach compared to our normal OPT practices. We also considered the difficulties, threats, or obstacles, and what positive experiences we could see...Having those discussions helped clear up misconceptions and false beliefs, and now I believe it’s working much more effectively." OPT22PC2Y |
| Emotional responses to increased understanding (concern, anxiety) | Worry of the patients fear-avoidance behaviour (0) | "And then also, I have become concerned when a person says they’re afraid of doing something, that it’s really limiting them, and they’re worried whether it’s serious and feel they should be referred immediately or wonder why their back isn't being imaged. We’ve been having these kinds of discussions more often during consultations than before." OHP10PUC2N |
|  | Anxiety when patient misunderstands HCP’s words (0) | "I actually cried after one client appointment when I had said that you can use your back despite the pain... The client really cling those words." OPT8PC4Y |
| Professional satisfaction, sense of adequacy and calmness | Having a sense of peace (+) | “I don't feel like a bad physiotherapist if I can't get everything done in one session. If the first session is mostly spent on discussion, we can still get far with that, and it can give me a sense of peace. And then it helps in building trust with the client, making them believe that there will be progress.” OPT11PUC2Y |
|  | Feeling of doing the job well (+) | "I was just thinking about a similar experience as [anonymized], paying more attention to the individual encounter and being quiet so that the person can talk. Somehow, it has positively brought something new to my own work. When you truly meet the person and have the conversation, you also get a lot out of it yourself. It leaves you with the feeling that you have done the job well." OPT20PC2Y |
|  | Feeling of adequacy as a professional (+) | "So probably listening to the client has become even more emphasized after this training and asking questions so that the client brings up things. And then showing the client that you understand them, realizing that you are not a bad OPT even if you don't give them a bunch of instructions." OPT11PUC2Y |
|  | Natural and easy to encounter patients (+) | "...difficult things, maybe I haven't dared to open them up myself, thinking that it's better not to stir things up. It feels like I didn't necessarily have the means to handle them, or on the other hand, I didn't dare to open them up much. Now it's not about holding back but rather asking a bit more. Somehow, it feels like the conversation flows more naturally now." OPT21PC2Y |
| COM-B=Capabilities, Opportunities, Motivations, Behaviour -model; BPS=biopsychosocial; HCP=healthcare professional; LBP=low back pain; MRI=magnetic resonance imaging; OHS=Occupational health services; OHP=occupational health physician; OPT=occupational health physiotherapist; OHN=occupational health nurse; TDF=Theoretical Domains Framework; | | |

**Table 1b: Capabilities, opportunities, and motivations for the systematic use of risk stratification tools at an early stage in the assessment of patients with LBP**

| SUBCATEGORY | OPEN CODES    (+) = facilitator  (-) = barrier  (0) = neutral | Quotation translated in English |
| --- | --- | --- |
|  |  |  |
|  |  |  |
| Knowledge and ability to use the tools | Understanding the meaning of the tools (+) | "There are also these questionnaires that assess the likelihood of back pain prolongation." OHP10PUC2N |
|  | Non-awareness of the tools  (-) | "Well, first of all, [anonymized OPT] gave information about this research in our internal [unit] meeting. This was done through Lync. I also presented the topic in at least one OHP meeting, if I remember correctly. During this, we were supposed to distribute an operational guide, and I think it is available to HCP electronically, but I suspect it hasn't reached everyone." OHP8PC4Y |
|  | Nonfamiliar with the stratified model of care (-) | "And we do have quite a few new OHNs, but as far as I know, we haven't really been in that kind of training. We've received information that something like this is ongoing, but I wouldn't say it's directly a treatment model because we don't know exactly what the model is, or how it works. At least I can't say precisely what the treatment model is. But of course, we know what is being discussed and so on." OHN7PC2N |
| Using tools to clarify LBP complexity and workability | Gives a lens to something that is difficult to describe in words (+) | "It's some kind of effort to get an objective measurement on a scale for something that's hard to describe in words." OHP7PC4N |
|  |  |  |
|  | Important factors related to pain problem and workability can be discussed with their proper names (+) | "In my opinion, these are really useful and important things when we consider the work capacity of people with back problems, and that we get to talk about these issues by their proper names and can take into account all the factors that are associated with LBP problems." OHP2PC1N |
| Conscious decision to apply the tools systematically | Systematically using the tools in workability assessment (+) | “My wish is that we would at least use it systematically for those people who at my request go for a work capacity and functional capacity assessment to [anonymized OPT]. At least that's how we have systematically used these." OHP2PC1N |
|  | Using the tools as a homework for the next appointment (+) | "But they can bring it up the next time that we meet… If you just give them the instructions, like 'take this and think about it at home, and then we'll discuss it next time." OPT17PC2N |
| Conscious decision for applying the tools to selected patients | Using the tools with new patients (0) | "The use of the SBT questionnaire scores has been quite limited recently, likely due to the fact that the clientele doesn't change much. When the study began, we systematically did it for everyone. But now, many of those who come for back issues have already been through it. For new clients, we go through everything at least verbally. At least in my own practice, I notice that I continuously assess these things and fill out the SBT questionnaire as needed if risk factors seem to be highlighted. So, it is still in use." OHP1PC1Y |
|  | Using the tools with patients with persistent LBP (0) | "Well, I have now started using the ÖMPSQ-SF questionnaire for chronic cases—not acute ones. But especially for clients who have seen me previously, perhaps a couple of years ago for a back issue and are now returning. Also, in those work disability cases where LBP is impairing their ability to work. I have systematically used the ÖMPSQ-SF in these cases, and I find it to be a very useful tool for assessing work capacity." OPT4PC2Y |
|  | Choosing from a variety of available questionnaires (0) | "It's individualized, you can surely find everything on the intranet, dozens of different questionnaires. These include mood assessment, anxiety assessment, pain experience scales, and functional ability scales." OHP4PC2Y |
| Conscious decision not to apply the tools at early stage | Experience of tools not always suitable for patients with acute pain (-) | "During the patient recruitment phase in the research, when it was filled out, it somehow always fit the situation very well. But my view is that for a lumbago patient who has been sick for a day or two, it is not used." OHP19PC2Y |
|  | Experience of need to build up trust before discussing about psychosocial issues (-) | "Well, of course, some of them have already filled it out when they've seen the OHP, so it's already completed, and I can just review what they've answered. And some clients, when they look at these questionnaires, feel that the questions are quite personal, and in some cases, I've noticed that the client might not immediately want to discuss everything... No one probably wants to appear weak, especially when you're in pain or have limitations, and you've had to give up certain things in your life. These can be quite sensitive areas. So, you don't always dive straight into the tough topics right away; you first need to build trust with the client." OPT1PC1Y |
|  | The HCP’s preference to interview instead of using the tool (-) | "I ask the same things, or the same issues, and I write them down in the text... Just my opinion, that by having a conversation, you get more out of the person. Instead of quickly talking about something and explaining, I do ask the questions, but I don’t use the questionnaire." OPT18PC2N |
| Memory and decision making of using the tools in clinical routines | Assessment of patients' body control more important in the OPT’s work (-) | OHP2PC1Y: "Well, maybe since I've been doing this job for over 30 years, it's not just this form that makes me aware of something." OHP2PC1N: "Hmm." OHP2PC1Y: "Even just the mental health aspect, I might not focus on that side particularly, but it's been quite interesting to use this form and see when it reveals something. I've been surprised that the risks are often quite low, even though I might see a higher risk in the person's behaviour or body usage. Sometimes I think a certain area might become even more problematic." |
|  | The evaluation of medication and sick leave more important in the OHP’s work (-) | "I've sent clients to [anonymized OPT] and thought that [anonymized OPT] would then talk and take care of things. I've somehow thought that my job is to assess whether they should continue working, whether I should write a sick leave, what medications they might need, and whether or not they should be operated. Those kinds of assessments need to be made, so I don't know, it hasn't really brought about much change in practice." OHP11PUC2N |
|  | HCPs experience the tools too complicated to use (-) | "Maybe that is exactly the barrier to why they are not used systematically because they are also a bit challenging for oneself." OPT14PC2Y |
|  | Not remembering how to score the tools (-) | "They are not so handy; there should be two questions, yes or no, and that would be it. These are so broad, and if you then have to calculate some index right away, with some having positives and negatives [scorings]." OPT17PC2N |
|  | Forgetting to use the tools (-) | "Now it's kind of forgotten again, but still, those principles are in my mind..." OHP19PC2Y |
|  | No time to use the tools systematically, and mark the scores to patient records (-) | "It's just that in the consultation, people might have many issues at once, so there really isn’t enough time. And it’s not just that you would start going through things systematically and writing them down, asking them to fill out the scale and then scanning the results. But in principle, you try to assess it, but less of that now." OHP5PC3N |
|  |  |  |
|  | Clear process of HCPs’ of using the tools and marking the scores to patient records (+) | "I rarely use ÖMPSQ-SF right at the beginning [of the appointment]. Asking someone to fill it out immediately makes me feel like I lose the opportunity for connecting with the patient and listening their perspective. This is just my way of working. I might talk and engage for a long time first, and usually, if I’m looking for some exercise instructions or lifestyle advice or if we have those relaxation exercises... When I’m printing that out or giving the next follow-up time, I’ll say, 'Fill this out while I’m looking for it.' And then they fill it out. At the same time, I might glance at the risk score and mark it for the next time. I usually document the risk in our patient database and note the most important things that stand out. That way, they serve as the basis for discussion at the next visit if they weren’t already during the first visit." OPT22PC2Y |
|  |  |  |
|  |  |  |
| Team agreement how and when apply tools | Agreement of the work distribution among professionals of tool use (+) | "We have considered how we manage our internal work distribution [in OHS team] and what kinds of psychosocial risk forms OPT can use more regularly with the clients, for example, those I refer for workability assessments. And this kind of discussion has probably been carried out more systematically, at least partly due to this study." OHP2PC1N |
|  | No team level agreement (-) | OPT25PC4N: “But we probably don't really have a common agreement  OPT23PC4Y: We don't OPT25PC4N: It is that, well OPT23PC4Y: Yes OPT25PC4N: Which could indeed be quite good to have" |
| Organizational policies guiding tool use | Tools included in the organization’s electronic questionnaires (+) | OPT6PUC2Y: “In the triage these are used online... And then the customer service desk people do the same, the nurses there OPT12PUC2Y: They ask the same questions as in the SBT” |
|  | No organization level agreement how to use the tools (-) | "The practice has probably been very, very varied, and most often it has been stated that there are no acute red flags or immediate imaging indications, so we refer to OPT. That's how it's most often documented, but there aren't necessarily any scores systematically documented." OHP7PC4N |
|  | No possibilities to influence on organizational level decisions considering policies to use the tools (-) | "I don't really know myself [if questionnaires can be added to the electronic questionnaires] because all of this kind of goes beyond our influence." OHP9PC4N |
|  | No policy on marking the scores to electronic patient records (-) | "It probably hasn't been systematically documented in any way. There hasn't been any agreement about it." OHP7PC4N |
| Patient responses influencing professional behaviours and decision-making | Patient does not find the questions of the tool relevant (-) | OPT14PC2Y: "And then many of those who didn't have pain related fear or in general felt that... when they started answering, they didn't get anything out of it." OPT19PC2Y: "They went too psychological. They went too deep, maybe?" OPT14PC2Y: "So, so they felt that they didn't have such issues, and they didn't feel like they wanted to engage with them. There were such experiences, meaning they didn't feel that they were psychologically or physically broken to the extent that those questions were relevant to them." |
|  |  |  |
|  | Patient questions the need of the tool (-) | "In my opinion, at least, the ÖMPSQ-SF is such that during this study when I tried to have people complete it, many felt it was too... that they didn't feel like they had... many were left wondering, 'Hey, I can't answer this. How does this relate? Why?'" OPT14PC2Y |
| Social and system-level influences encouraging tool use | PRM specialists require the use of the tool before consultation (+) | "And specifically, the PRM specialists want us to use these questionnaires. ÖMPSQ-SF is needed, they systematize it, I know that now." OPT19PC2Y |
|  |  |  |
|  | Insurance companies prefer the use of measurements for assessment of work disability (+) | "So, this is mainly in prolonged workability cases when the insurance company requests these metrics and figures, in those cases. But certainly not for every pain patient, not really. But in risk cases." OHP4PC2Y |
| The tools serving as conversation starters | Aids conversation (+) | "I've also used those questionnaires, and it somehow feels like they've been very useful. If a client doesn't start to produce much on their own, it's like a trick that somehow prompts them to think a bit more broadly about their pain situation as a whole, and then it easily moves the conversation forward." OPT10PUC2Y |
|  | Allows to ask about difficult issues because the patient has produced the information (+) | OPT24PC4N: "I actually use them [the questionnaires] also because it's easier for me to start a conversation about some difficult topics. In that case, I'm not the one bringing up the issue." OPT9PC4Y: "Mmm." OPT24PC4N: "But when we look at the form, I can say, 'You filled this out here, what does this mean to you?' and so on. It allows me to ask about difficult things more naturally because the client has already mentioned it on the form." OPT25PC4N: "Yes." OPT24PC4N:"That's why I like them because they often serve as conversation starters for me. Those forms." OPT25PC4N: "That's good." |
|  |  |  |
| The tools aid in clinical reasoning | Awakens to consider the patient’s situation as a whole (+) | "But then, the questionnaires have concretely helped me; they have been good aid... And somehow, it makes it easier to look at the situation more holistically and I don't feel so helpless. OPT12PUC2Y |
|  | Helpful in clinical reasoning (+) | "It's exactly this kind of clinical reasoning as well; you can make a much better action plan when you actually use that form." OPT25PC4N |
| The systematic use of the tools improves treatment planning, quality of work and saves time | Ensures the treatment line is considered in advance (+) | "At best, it then goes so that it has already been done by the time it is directed to the physician, so it would change then... So that it is directed to the right place, and the questionnaire can serve as one way to guide the patient to the right place." OHP9PC4N |
|  | Enables making action plan at early stage (+) | "Maybe that's where the change lies, in how I also guide the initial stages of physiotherapy. In those discussions, we go over the plans and use the SBT to identify any risk factors that arise, addressing them differently and planning the future visits more thoroughly. So perhaps the change is that I haven't just referred the patient to an OPT for assessment and guidance but have already thought through the content of those visits in a more planned way." OHP1PC1Y |
|  | Systematic mapping of psychosocial factors improves quality of work (+) | "That is a matter of quality in our work, to ensure that they [evaluation of psychosocial factors] don't get forgotten in the rush." OHP2PC1N |
| Establishing goals for systematic use of the tools | Individual goal to use the tool systematically for risk stratification (+) | "And then probably the fact that we would consciously start to identify factors related to the risks of... Prolongation... And not just from the severe situation, but from every case. What is the risk that this might become recurrent or prolonged or continuous? Maybe we haven't done that so consciously before." OPT9PC4Y |
|  | Opportunity to guide the direction and extensiveness of the rehabilitation process (+) | "I think about it in terms of patient risk assessments and stratification, in a way. I just got the idea that the SBT could be a fully digital tool used before the appointment, which would somewhat guide the direction in which the patient is steered or how intensive the interventions should be at different stages of our process." OHP14PC2Y |
| Organizational goals supporting clinical practices | Goal to apply systematically in the organization through electronic questionnaires (+) | "And currently, for our OPTs, we are in the process of implementing the ÖMPSQ-SF. We aim to make it almost systematic, and the idea is that at some point it will be sent electronically to the client before their appointment. The goal is to have it filled out in advance, and we hope to have this implemented sometime this year. That would significantly ease its use." OPT21PC2Y |
|  | Training HCPs nationwide to facilitate the systematic use of tools in organization (+) | "In principle, this [ÖMPSQ-SF] is used for every patient, and this is how we have trained our OPTs nationwide. But it’s not quite routine yet, as it tends to be forgotten." OPT22PC2Y |
| COM-B=Capabilities, Opportunities, Motivations, Behaviour -model; TDF=Theoretical Domains Framework; BPS=biopsychosocial; LBP=low back pain; HCP=healthcare professional; OHP=occupational health physician; OPT=occupational health physiotherapist; PRM=physical and rehabilitation medicine specialist | | |

**Table 1c: Capabilities, opportunities, and motivations for multidisciplinary collaboration targeting an individualized plan for patients with LBP**

| SUBCATEGORY | OPEN CODES  (+) = facilitator  (-) = barrier  (0) = neutral | Quotation translated in English |
| --- | --- | --- |
| Therapy skills to target individual goals | Targeting therapy to patient’s everyday functioning (+) | "Clients receive more practical exercises that they can commit to, and it's now rare to give purely therapeutic exercises for LBP." OPT10PUC2Y |
|  | Patient’s individual goals involved in the treatment plan (+) | "Just that, to continue, it should be more focused on the client’s specific needs and the difficulties they face in their daily life and work. It should go to the core of it, rather than just giving some strengthening or stretching exercises. We need to identify the real problem that is hindering them in their everyday life or work and address that. It should be a more participatory approach, listening more to the client so they can bring up the solutions themselves and say what they are willing to do. My role is to then support those actions.” OPT12PUC2Y |
| Confidence to take responsibility for the treatment process | OHP’s courage to take responsibility of the treatment line (+) | "Probably during this research trial, confidence in one's own skills has increased in the sense that, when considering referring elsewhere, there’s a feeling that one can take more responsibility for the treatment and follow the situation for a longer period." OHP1PC1Y |
|  | Trust in multidisciplinary team capabilities before referring the patient to imaging (+) | "When a client often says that they have already visited [OPT] in this or that year, I try to motivate them by explaining that we now have completely new system. I share examples of my own patients who have spent half an hour with the physiotherapist and walked out pain-free, standing straight. This shows how quickly we can achieve relief. I have had very good experiences where we have implemented this system with [anonymized OPT] and seen remarkable recoveries. This allows us to avoid unnecessary imaging when it is not needed." OHP18PC2N |
|  | Patience to see the results of the rehabilitation before referring to specialized care (+) | "When there is nothing but a non-specific status, we can manage it here for a longer period. And the longer we manage it, the higher the likelihood of improvement without a specialist visit." OHP19PC2Y |
|  | OPT's confidence of being capable of treating more challenging patients (+) | "I gained a lot of confidence from the [place of BPS education]. For example, before, I would quickly refer patients with radicular symptoms back to the OHP to consider an MRI. And okay, many of my client companies have very good contracts, so it was easy for them to get an MRI, and it still is, but now I definitely keep the threshold high. Radicular symptoms don't make me anxious anymore. It almost has to be foot drop or leg giving out before I refer them back to the OHP. So yes, it definitely had a direct impact on me." OPT4PC2Y |
| Decision-making and prioritization of OHS resources | OHP's decision to use all needed multidisciplinary resources in the unit (+) | "But generally, most OHS clients can be managed with a multidisciplinary team and the methods we have available. However, for cases where there is a prolonged issue or a very complex overall situation, the support of the entire multidisciplinary team is needed at different stages of the process." OHP14PC2Y |
|  | OPTs not part of decision process of referring the patient to specialized care or use of multidisciplinary resources (-) | "The client working groups that I'm part of, where I'm now only involved once every six months, don't deal with individual cases. It's more focused on workplace-based issues and building frameworks related to organizational and collaborative matters, not individual cases. So, I haven't really had the opportunity to share examples of how I've worked with specific clients. It would definitely be useful to have those discussions, of course. In a way, I've just accepted my role in this." OPT13PUC2Y |
|  | The OPT experience of being an outsider of the OHS team (-) | "I sometimes feel a bit like an outsider in that regard, that it's mostly the OHNs and OHPs who are more involved. And then there's me, as a specialist—which I am—who is brought in as needed for certain things, if it seems necessary. So, we're not as much of a close-knit multidisciplinary team." OPT13PUC2Y |
| System level structural barriers for rehabilitation in OHS | The system does not sufficiently support early-stage rehabilitation in OHS (-) | "Well, these are such big societal issues, like the difficulty of using partial sick leave, and KELA [National Insurance Company] doesn’t really require any models for work modifications. Okay, there’s the 30-60-90-day rule, but nothing for earlier stages, and for partial sick leave a statement is required. It’s not enough to just tick a box like in Sweden, and keeping someone on partial sick leave can often mean they get more money staying at home than working part-time. And for direct access physiotherapy, there needs to be a referral from an occupational health professional [OHP/OHN]. These kind of seemingly artificial barriers exist because we’re in an OHS organization, as opposed to a health centre where you can just give the appointment to the physiotherapist without worrying about who pays." OHP2PC1N |
|  | System level policy not allowing more than three OPTs’ appointments in Finnish OHS (-) | "And then if you think about the protocol, where if you’re at a certain risk level, you should have a certain number of physiotherapy sessions, that doesn’t really happen in OHS where you get three, max five visits. Some even have only two." OPT14PC2Y |
| Variability in practices based on client company size | Handling small client companies in the unit/team (patient follow-up difficult to organize due to costs, no regular team meetings) (-) | "We are a small unit, so I have this feeling—because of my experience with having clear teams where individual client matters are discussed—that here, we haven't had designated time for that. So, I often hear things, incidentally, like in the coffee room, but that is probably due to the small size of our client base. There might not be a payer for the common meeting where these matters would be discussed." OHP2PC1Y |
|  | Large companies (rigid, not possible to create practices locally) (-) | "In these large government organizations, there is a lot of rigidity. These kinds of issues have a greater impact on sick leave practices. We have large companies where, if you write a sick leave recommendation for a cold or even back pain for a few days and tell the person they can go back to work a day or two earlier if they feel up to it, they can't because they have that physician's recommendation. And when we discuss contracts and such, I always try to highlight the possibility of a self-notification practice [for sick leaves]. I think it's an important issue because it makes the concept of sick leave more flexible and natural for people. It means they will have to struggle less with this issue here." OHP2PC1N |
| Physiotherapy and rehabilitation resources within the unit | Direct access to physiotherapy without delays (+) | "We have a customer promise that an acute [OPT] appointment is available within 1-3 days, and usually, it's available on the same day. OPT22PC2Y" |
|  | Long wait for patients to OPT (-) | "Our schedules were already blocked for a long time; we didn't necessarily get any new clients, but rather continued with the ones we already had." OPT24PC4N |
|  | Flexibility to use OPT according to needs (+) | "In practice, the amount of occupational physiotherapy depends on how well the guidance starts to work, and we aim to provide guidance to everyone who needs it." OHP1PC1Y |
|  | Insufficient resources in the unit for BPS trained OPTs (-) | "Well, at that point, we had a significant shortage of resources. We had a very challenging situation, with probably a shortage of about three OPTs at that time." OPT24PC4N |
| Collaboration outside OHS to ensure active rehabilitation | Collaboration with BPS trained HCPs outside OHS (+) | "Indeed, when referring a client for physiotherapy outside of our organization, we have a practice that the client can choose which therapist they go to. But sometimes the client asks for recommendations, like which therapist would be best for their symptoms. These can be challenging situations. And [sigh], I always hope that the colleague can continue from where we left off. It’s beneficial since the client is also investing their own money into it. Of course, there is some assistance, but we could still build more collaboration if we had more time resources to work with these colleagues. I notice in my everyday work that if a client has seen a physiotherapist who understands pain and considers the client comprehensively, I see positive effects in the client's endurance and ongoing well-being." OPT1PC1Y |
|  | HCP cannot influence on the content of rehabilitation outside OHS (active/passive) (-) | "If a company covers, say, 5 or 10 physiotherapy sessions, some may have specified whether it's with [OHS]'s own physiotherapist or a partner [outside OHS]. But if there are no specific guidelines, then a client might end up using all 5 sessions for massage. It's a bit like, maybe it should be clearer what the follow-up should be, that it should be active rehabilitation." OPT15PC2N |
| Access to new tools for individualized treatment planning with patient and a multidisciplinary team | Using risk stratification in multidisciplinary treatment planning (+) | "Yes, we try to ensure that when we identify an individual at risk, we cycle them through OHN, OPT, and OHP. It's good because the questionnaire might indicate the need for a psychologist as well." OHP2PC1N |
|  | OPT using Patient-Specific Functional Scale for identification of functional goals together with the patient (+) | "We have the clinical reasoning form that we use. where we fill out the blank form. It includes, for example, the Patient Specific Functional Scale... Mainly it focuses on the functional impairment and what in the work environment is causing that functional impairment." OPT8PC4Y |
|  | Importance of including goals for leisure time, not only work-related functions (+) | "I have used the Patient Specific Functional Scale, meaning the goals where pain has prevented certain activities. We look at what goals we can start working towards. There is a clear plan there. It hasn’t always been about work. Quite often it has been, but there are many things in leisure time that one wants to achieve that he/she hasn’t been able to." OPT22PC2Y |
|  | Making the rehabilitation plan based on risk stratification in collaboration with all team members (+) | "And then we have weekly multidisciplinary meetings where we discuss things, and if any concerns or high-risk issues come up, we then make plans for how the care will proceed." OPT1PC1Y |
| Influence of authorities in treatment planning | OHP with authority helping to convince the patient for the treatment plan making it easy for OPT to proceed with rehabilitation (+) | "I think I've received a lot of help because when [anonymized OHP] has talked with the client, I then get help from knowing what words you used and whether the client still has any concerns. This, in turn, makes it easier for the continuation and guidance." OPT1PC1Y |
|  | Orthopaedic surgeon consultations provide non-individualized treatment plans for rehabilitation (-) | OHP13PC2Y: "When I talk about the [anonymized organization] policy, we are in quite a bind if they [patients] are sent to an orthopaedic surgeon or somewhere, and then he asks for the imaging, and then..." OPT17PC2N: "It may not align with the approach of functionality and self-directed rehabilitation." |
| Creating continuity of care through organized follow-ups | OPT creating trust by giving direct phone number for patient (+) | "I always give my phone number so that if you have any doubts, flare ups, or problems, you can call me. Very rarely do I receive calls. This one male I mentioned has called me several times. He has said that it has been an important part of his treatment—to always be able to reach me and get that reassurance, help, and relief from worrying. I think the call is important because it means the person doesn’t have to worry. They know they can always reach someone quickly." OPT22PC2Y |
|  | Organizational policy to contact the patient after the OPT appointment by phone (+) | "Many clients give positive feedback during appointments, appreciating that someone is interested in them and that the OPT called to ask how they are doing. This is a standard [follow-up] practice at [anonymized OHS organization]." OHP4PC2Y |
| Systematic monitoring of treatment processes and case management of high-risk patients | Systematic company level monitoring of sickness absence rates and patients with M-diagnosis are contacted by OPT (+) | OPT4PC2Y: "Now we have new electronic tools, like these compasses and others, which automatically give a signal to the OHN about certain prolonged absences. The nurse checks if there are any issues, and then it moves to the OPT, who contacts the patient to check on their condition and possibly schedule follow-up actions. So, this is well taken care of at our end." OHP4PC2Y: "Similarly, we monitor sick leaves for companies monthly or quarterly, and all employees in the M-group are directed to OPTs. The OHS contacts them to see if the treatment has started." |
|  | No organizational agreement of care pathway or active monitoring of high-risk patients (-) | "And probably for these high-risk patients, based on the questionnaires and interviews that identify them, we don't have a care pathway available to follow for these cases." OHP1PC1Y |
|  | Nurse / physician responsible for the care pathway for high-risk patients (+) | "The practice and principle here is that if it seems necessary, if there is a high-risk client, they are referred to a OHP or OHN. They then consider the treatment path and refer it further." OPT22PC2Y |
|  | No case manager for high-risk patients (-) | OPT17PC2N: "So, if we want to, there is a clear need and desire to develop this type of activity, but then there must be a need and the timely opportunity to implement it." OPT16PC2N: "And a person to coordinate it." |
| Available care pathways and options for high-risk patients in the unit | Clear process of stratified care agreed in the organization (+) | "If it's possible, as it often is, the SBT is used during the appointment to score and categorize individuals into low-risk, moderate-risk, and high-risk groups in terms of their risk of developing chronic non-specific LBP. Based on this risk stratification, low-risk patients are directed to an OPT, especially if they have strong beliefs or recurrent low back pain. Moderate-risk individuals are also referred to an OPT, and high-risk individuals are assigned to OPT trained in this new approach, namely [anonymized, anonymized and anonymized OPTs]. If stress or sleep disorders are identified, we consider how occupational health psychologist can be utilized in their treatment. Since this was part of a patient recruitment model for the research, patients were informed that a LBP study was ongoing, but regardless of participation, treatment would follow this practice. We also ensured that this approach was consistent with the Finnish Current Care -guidelines." OHP8PC4Y |
|  | Non-existing or unclear LBP rehabilitation processes (-) | "In my opinion, some OHPs still prescribe long sick leaves and just give a prescription without considering us [OPTs] at all. But fortunately, a large part of them do remember us." OPT11PUC2Y |
|  | Psychologists not actively part of treatment of patients with LBP (-) | OHP9PC4N: I think it's still in its infancy here. OHP8PC4Y: Pain psychologist. OHP9PC4N: I started thinking about whether it is very common for psychologists to be involved in the treatment of musculoskeletal symptoms here. OHP8PC4Y: No, it's not common at all. OHP9PC4N: Now I started thinking that... OHP7PC4N: It has to be quite a terminal phase for psychologists to be involved with retirement issues. |
|  | Starting BPS multidisciplinary group rehabilitation in the unit (+) | “And we have also incorporated this [BPS approach] into group sessions.” OPT11PUC2Y: |
|  | No multidisciplinary group activities in the unit (-) | "We have thought about pain groups, but they have probably never even been organized." OHP9PC4N |
|  | Rapid multidisciplinary collaboration for high-risk patients (+) | "When the first three [OPT] appointments were completed, and I had divided them into half-hour segments. We have a system where costs cannot increase, but the number of visits can be higher. So, as I told the OHP, I asked if I could get more, if I could get three more sessions. And the OHP said, of course. So, I divided those into half-hour segments as well, meaning the patient has now had six or even nine sessions. And there are still three new sessions left. And then there's also the [group] on top of that. So, we have ways to continue supporting the patient if they are a high-risk case. And we even received feedback from this patient, who also told the physician that this was the first time OHS has worked in such a multidisciplinary way, reacting immediately and making contact, whether it's the OHP reaching out to me or me suggesting a psychologist. The patient was really pleased. It was a completely different experience from before." OPT22PC2Y |
| Organizational and team stability ensuring continuity in multidisciplinary work | Regular interprofessional meetings ensuring continuity of care (+) | OPT1PC1Y: " We also have these weekly multidisciplinary meetings where we go over everything." OHP1PC1Y: "In practice, both OHNs participate in these meetings, and during them, we also discuss other long-term challenges and sometimes even short-term issues that need to be addressed together. Specifically, if something related to LBP issues comes up—like changes in behaviour or even just the general planning of a rehabilitation program—we openly discuss it with the whole group, and everyone can share their opinions." OPT1PC1Y: "Our multidisciplinary collaboration is so close-knit because we're a small unit, so we don't always even think about it—it just feels like everyday life. But it really makes the work much easier since everyone working with the client is aware of what's going on and what needs to be done next." |
|  | Multidisciplinary collaboration based on knowledge-transfer through electronic patient records (-) | "And unfortunately, collaboration is somewhat limited because there simply isn't enough time; everyone works independently. Information is mainly conveyed through documentations in medical records." OHP4PC2Y |
|  | Patients are treated by many different HCPs with high turn-over and short work periods (-) | "You know, to get our physicians on board. We just talked about how there are some OHPs who are very much aligned with this way of thinking. But then we have quite a few physicians who come and go, they are temporary, doing shifts or staying with us for a short time, and even they, even they need to get on board with the communication so that we find a common language. It's a very challenging situation to make this work in a large unit for everyone." OPT23PC4Y |
|  | No time or possibilities for multidisciplinary implementation meetings due to financial reasons (-) | "When each of us works as a private practitioner, time is money. Most are working hard with dollar signs in their eyes." OPT5PC3Y |
|  | Changes in job descriptions (-) | "Well, our problem has probably been the high turnover in the unit where I work. We had an OPT who was involved in this but resigned. That was probably over six months ago. OPT changed jobs and couldn't commit to this as expected." OHP3PC1Y |
|  | Flood of information (digital leap) (-) | "It's probably that over the past two years, we've been in the midst of so much change in this organization. There's just so much information flow." OPT11PUC2Y |
|  | Reforms in the organizations (-) | "Probably one reason is that there were so many other changes happening at the same time, so they couldn't really absorb this additional change. In our company, we constantly have new practices and procedures being introduced, so this was by no means the only change." OHP3PC1Y |
| Interprofessional identity and shared knowledge of BPS management | Multidisciplinary BPS training involving both OPTs and OHPs from the same unit/team (+) | OPT6PUC2Y: “But it was good that we had the physicians involved. OPT11PUC2Y: Indeed. OPT6PUC2Y: If they hadn't been there, it would probably have been quite difficult” |
|  | Interprofessional group identity and peer support of BPS trained PTs and physicians (+) | “Just as we have been discussing these things among us, I feel that we have quite a common language and shared understanding of this.” OPT23PC4Y |
|  | Disconnection between BPS training and routine OHS teamwork (-) | "And of course, I see that [BPS] training is one of those things that really opens up the topic in a certain way. I see the importance of training, and it would have been better if it had been available to a broader group. The only downside was that it was held in [anonymized place], which was quite far from our perspective. It was really challenging to get people to attend. It would have been really important for that training to be conducted within our own unit, where we could have started implementing it. And the team that would be responsible for carrying it forward would have been involved. In that sense, these activities were somewhat disconnected, and that likely influenced the outcome." OPT23PC4Y |
|  | Sharing knowledge of BPS approach with interprofessional meetings (+) | OPT11PUC2Y: “Well, [anonymized OHP] has been involved a lot in customer service, and we've even held our own information sessions there. OPT10PUC2Y: Mmm. OPT11PUC2Y: And then we have a lot on the intranet, and we've discussed it in professional meetings and in [multidisciplinary] teams. OPT10PUC2Y: Everyone in their own teams. OPT11PUC2Y: Regularly. OPT10PUC2Y: And working in client work groups, when there are individual client cases, they share a bit about what has been done and what has been discussed with the client, and through that, they ask more about what this is all about. OPT6PUC2Y: It's probably also discussed at the coffee table in some informal chat circles, where this information gets shared with someone, at least with an OHN who might have a patient like this, even with the OHP.” |
|  | Influential HCPs facilitating the interprofessional knowledge transfer (+) | OPT11PUC2Y: It should be noted that [anonymized OHP] has attended the OHP meetings at least once or twice, providing information there, and [anonymized PRM specialist], has also been involved. OPT6PUC2Y: [anonymized OHP] has also been [sharing information] in the coffee room. OPT11PUC2Y: Yes, and he also informs the clients. |
|  | Continuing BPS education in the organization to unify and harmonize the treatment pathways and multidisciplinary collaboration (+) | "I do believe that unifying our approach and internal [BPS] training so that we are on the same page, even though the clients are different, helps to standardize and harmonize the care pathway and our multidisciplinary collaboration." OPT15PC2N |
|  | Challenge of leading others in BPS approach (-) | "It's quite difficult to go into the room and supervise, like forcing someone to do this. This is such a topic that it somehow needs to be internalized and felt as one's own in order to do it successfully." OHP3PC1Y |
|  | Challenges in sharing of knowledge and experiences within the large unit (-) | “Maybe there could have been people chosen for [BPS training] who are directly involved in the practical work. That way, it would integrate better since they know what they are doing. For instance, [anonymized OHP] was no longer doing practical work at the time; she was trying to transfer that knowledge to the OHPs and its significance, which was quite challenging.” OPT23PC4Y |
| Expanding role, confidence and boundaries | The role of OPT more versatile, not focused only to ergonomics (+) | "If there have been our OHS team meetings in the unit, maybe I more boldly bring up my expertise in a broader context. When we are considering the issues of a client company, we don't just limit OPT to the physical factors or physical ergonomics but consider everything it encompasses. I think it has been very fruitful, leading to many good discussions, and my utilization has clearly increased as a result." OPT9PC4Y |
|  | The OHPs experience that the role of the OPT is emphasized in relation to pain behaviour, fears, and treatment of LBP (+) | "Perhaps then in pain management, pain behaviour, and pain-related fears, the role of the OPT has been emphasized." OHP1PC1Y |
|  | OPT confidence to bring out the expertise more broadly in multidisciplinary collaborative situations (+) | "Perhaps through the effectiveness of multidisciplinary collaboration, we have achieved more. At least I feel that way and see the changes compared to earlier times. It gives one the confidence to speak up and understand one's role, knowing what can be recommended." OPT5PC3Y |
|  | Positive patient experiences of OPT applying BPS approach with patients with both mental and musculoskeletal issues (+) | "I suggested to the client that perhaps they could continue discussions with a psychologist, an occupational health psychologist, about these issues with coping. They preferred to come here rather than go there." OPT23PC4Y |
|  | Psychologist not always needed when OPT applies BPS approach (+) | "It is possible to refer clients to an occupational health psychologist through the OHP. I have also considered using the psychologist as a collaboration, but I haven't done it yet. But I will try it; currently, there hasn't been a need. I don't see the point in forcing it. Somehow, the clients have been so happy with this BPS approach that it has already opened some good things. So, I haven't needed the occupational health psychologist yet.” OPT4PC2Y |
| Increasing physiotherapy use and improving patient outcomes and return-to-work | Increases use of physiotherapy (+) | Well, I've had the feeling that I've always referred a lot of clients to you OPTs, and maybe that has increased even more now that attention has been paid to it. And perhaps the approach of the OPTs has also become more focused on the psychosocial side. I have found that nice to notice. OHP3PC1Y |
|  | Accelerates patients’ return to work (+) | "I have a feeling that with this approach, we can encourage the client to return to work." OPT22PC2Y |
|  | Decreases number of patients waiting for specialized care (+) | "I think it [the number of patients referred to specialized care] has decreased. Since this has worked so well, I don't see any reason to go to specialized healthcare. It must have decreased." OPT22PC2Y |
| Enhancing multidisciplinary collaboration | Multidisciplinary collaboration more effective than before (+) | “Definitely, it has lowered the threshold for all professional groups to send emails about clients or to discuss things more richly and multidimensionally in joint meetings. That's how I feel.” OPT9PC4Y |
|  | Increases referrals between HCPs (OHN-OPT-OHP-psychologist) (+) | "If there's a need of OHN or something that would require a psychologist's visit, we have had cases referred from the OPT to the OHP, and through them, they have gotten a referral to the psychologist. It has happened that this professional group has also been referred because of this." OPT20PC2Y |
|  | Increases OHN confidence in referring LBP patients more quickly to an OPT instead of OHP (+) | "It has certainly increased our confidence in how quickly we can refer a patient with LBP to an OPT, whether the LBP is affecting them at work or during their free time. Indeed, the first point of contact doesn’t necessarily have to be the OHP, but rather the OPT." OHN7PC2N |
| Expectations regarding LBP treatment | Raising positive patient expectations towards multidisciplinary BPS management of LBP (+) | “Our [anonymized OHP], has really been pushing it forward, just like our PRM specialists who have supported the clients, and in this way, we have been able to convey it to the clients so that they are willing to try it out." OPT11PUC2Y |
|  | Avoiding early imaging due to low benefit and potential harms related to imaging (+) | "It has only reinforced that we are on the right track by not requesting lumbar spine X-rays because they are not beneficial and expose patients to a lot of radiation, which is not sensible anyway." OHP10PUC2N |
|  | Referring to imaging to support patient’s commitment to the treatment process (-) | "Given these experiences, whenever I get the feeling that imaging might be necessary, I do suggest it. It is possible to get imaging done privately so we can move things forward. Then we proceed with the imaging to see if there is a need for surgical treatment. I understand that it is easier to treat a very painful patient when you can say there is nothing that requires surgery. It makes the treatment process much easier, and the patient is more likely to collaborate with the treatment plan." OHP4PC2Y |
|  | OPTs experience patients prefer advice and active rehabilitation (+) | OPT11PUC2Y: And it seems that nowadays quite a few customers are fed up with only being given sick leave and pills. OPT6PUC2Y: Exactly. OPT11PUC2Y: Like when they have LBP, one came in recently, it was already the third week, and not once had it been suggested that they see an OPT. The customer had to ask themselves if they could go [to OPT], to get something they could actually do. |
|  | OPT experience patients to expect passive treatments even he/she prefers self-care and active exercise (-) | "I've been thinking about this because some companies cover [physiotherapy] treatments, some come and kind of demand to get that... referral again, like for massage. Then it becomes necessary to explain and justify it. I still need to figure out whether I should direct them there, how I should handle this, or if self-care is the important thing, or if physiotherapy is alongside that, and what physiotherapy entails. I've been thinking about that. [silence] Because people should still take care of themselves, exercise, and be active." OPT8PC4Y |
| Encountering HCP attitudes towards BPS management | Enthusiasm of having something new for the patients (+) | “Now I have been able to say, 'Hey, we have something new'." OPT11PUC2Y |
|  | Increasing interest of team members after successful patient experiences (+) | "And not everyone is interested. Some are interested. When some clients have received help from this, they notice it and ask about it, and their interest has then increased." OPT11PUC2Y |
|  | Buy-in the goal of finding patients’ abilities and opportunities despite the LBP (+) | "In my opinion, it's important not to focus solely on the pain problem but to seek out possibilities despite it or alongside it. In that this [BPS] approach has likely been successful as well." OPT17PC2N |
|  | Hunger for information of BPS approach (+) | "Quite a few [OPTs] became interested... during our training day on [anonymized date], interest was piqued. And they acknowledged that this won't be implemented just like that. Rather, a hunger for knowledge emerged, and I thought that was wonderful." OPT22PC2Y |
|  | Low enthusiasm and disbelief among multidisciplinary teams towards BPS approach (-) | "Well, especially from PTs and physicians and from personal trainers and everyone. And then when I go and tell about this, they look at me like I'm crazy." OPT5PC3Y |
|  | Shock, frustration and anger for not being able to use the skills from previous trainings (-) | "It was when we were there at [BPS education] for the first time, there were [anonymized number of OPTs from the unit]. It was quite a shock indeed, how all the previous things. We had manual therapists and McKenzie therapists. It was like, what about all of those? The first reaction was a bit of anger and frustration, and then about the ergonomics, the ergonomic guidance." OPT22PC2Y |
|  | Waiting for the research results from OHS context (0) | "...we are slowly moving towards this system, although the research evidence is still lacking, but this seems like a good system." OHP8PC4Y |
| COM-B=Capabilities, Opportunities, Motivations, Behaviour -model; TDF=Theoretical Domains Framework; BPS=biopsychosocial; LBP=low back pain; HCP=healthcare professional; OHS=Occupational health services; OHP=occupational health physician; OPT=occupational health physiotherapist; OHN=occupational health nurse | | |
