# Supplementary material for: Capabilities, opportunities and motivations in implementing guideline-oriented biopsychosocial low back pain management: perceptions of occupational healthcare professionals after an educational intervention
Source: BMC Health Serv Res. 2025 Aug 29;25:1153. doi: 10.1186/s12913-025-13267-7 (PMC12398078; doi:10.1186/s12913-025-13267-7)
Supplement: Supplementary file 3 — Supplementary Material 3 [file 12913_2025_13267_MOESM3_ESM.docx]

Appendix 1. Educational interventions

Initial biopsychosocial (BPS) educational intervention lasted 4 days (September 21-24, 2017) and booster training 3 days (June 3-5, 2018). Training consisted of live patient demonstrations, clinical case problem solving and role plays. The trainers were pain psychologist, professor Steven Linton, professor Jaro Karppinen and physiotherapist Kasper Ussing. Two experienced Finnish physiotherapists tutored the intervention study sites and provided supervision and feedback to support the learning of health care professionals (HCPs). The clinical champions (physiotherapists and physicians who participated in trainings) were educated to share the information at their workplace.

An online educational platform was created for the ‘clinical champions’, with scientific articles, videos, educational materials and a forum for questions. In the platform, HCPs had the opportunity to discuss educational topics. The ‘clinical champions’ were encouraged to share information in their own occupational health service (OHS) units more widely to those HCPs who were not involved in the BPS educational intervention. For this, they received a written educational material. After the training, at least one member of the research team visited each unit. During OHS outreach visits, the study design was explained, and the principles of BPS management of patients with low back pain (LBP) were briefly reviewed. During the on-site visits, practical issues related to patient recruitment and data collection were also discussed.

We offered HCPs evidence-based tools (patient education booklet, risk stratification tools (Start Back Tool (SBT) and a short version of the Örebro Musculoskeletal Pain Screening Questionnaire (ÖMPSQ-short) to assess the patient and implement treatment according to the BPS guideline used in the study (Figure 1). The assessment of patients with LBP taught in the training was multidimensional, including physical (ability to function), psychological (fear of physical activity, fear-avoidance behaviour, pain catastrophizing, pain-related self-esteem, depression), social (ability to work) and health-related quality of life components ^5-7^. HCPs were encouraged to systematically use the stratification tools in evaluation of the patient's individual risk profile. Physiotherapists were also recommended to use the ÖMPSQ-short.

Stratification of patients according to the SBT (Figure 1) was used as the basis of the treatment model: low-risk patients were mainly to be offered counselling about the BPS nature of the pain; moderate-risk patients were additionally recommended exercise therapy that improves functional capacity; and high-risk patients received psychologically focused treatment in addition to components of moderate-risk group. For all patients, the goal was to make the LBP understandable and to focus the guidance on observed risk factors according to the individual assessment. If necessary, the HCPs could refer the patient to, for example, an occupational health psychologist.

References

5. Tagliaferri SD, Miller CT, Owen PJ, et al. Domains of chronic low back pain and assessing treatment effectiveness: a clinical perspective. *Pain Practice*. 2020;20(2):211-225.

6. Lin I, Wiles L, Waller R, et al. What does best practice care for musculoskeletal pain look like? Eleven consistent recommendations from high-quality clinical practice guidelines: systematic review. *British Journal of Sports Medicine*. 2020;54(2):79-86. doi:10.1136/bjsports-2018-099878

7. Gatchel RJ, McGeary DD, McGeary CA, Lippe B. Interdisciplinary chronic pain management: past, present, and future. *Am Psychol*. Feb-Mar 2014;69(2):119-30. doi:10.1037/a0035514

Appendix 2. The interview guide

Semi-structured interview guide for focus group interviews in occupational health service (OHS) units. The interviewer explains the study purpose, the process and the rules for the focus group interview.

Introduction of participants (name, occupation).

**Question 1.** Can you tell me about your perceptions of the educational intervention on biopsychosocial (BPS) approach and whether the training has influenced on professionals’ actions when working with patients with low back pain (LBP)?

*After this question the conversation may flow freely. The participants are asked to tell more and encouraged to share their experiences of participating in the study and the assessment and the treatment of patients with LBP in OHS using following questions:*

Can you tell me more about…?

What do you think it is because of?

Previously you said that…

Can you explain how you mean

**Question 2.** Can you tell me about your perceptions on

- the respective (interviewed) OHS unit
- multidisciplinary collaboration

**Question 3.** Can you explain, how you mean by the BPS approach?

**Question 4.** What have been possible facilitators in implementation?

**Question 5.** What barriers for implementation have you experienced?

**Question 6.** Can you tell me about how have the professionals that participated in the BPS training implemented the information from the trainings in your team and the OHS unit?

**Question 7.** How many professionals actively use the BPS approach in your OHS unit? Could you define what it means for different professional groups?

**Question 8.** Could you tell me about the treatment pathways for different risk groups of patients with LBP – especially for patients with high risk of prolonged disability?

**Question 9.** Are screening instruments used systematically in your OHS unit?

**Question 10.** In your experience, has the educational intervention influenced on processes in your OHS unit?

- imaging practices in the unit
- the sick leave practices in the unit
- the referrals to secondary care

**Question 11.** Can you tell me about your experiences of the interaction with patients?

Appendix 3. Two-level categorizing matrix

The Theoretical Domains Framework (TDF) is an elaboration of the Capability, Opportunity and Motivation model (COM-B) model of behaviour with 14 domains of theoretical constructs that map on to the COM-B categories.

| **COM-B categories** | **TDF domain** |
| --- | --- |
| CAPABILITY | Knowledge |
|  | Skills |
|  | Memory, attention and decision processes |
|  | Behavioural regulation |
| OPPORTUNITY | Social influences |
|  | Environmental context and resources |
| MOTIVATION | Social/professional role and identity |
|  | Beliefs about capabilities |
|  | Optimism |
|  | Beliefs about consequences |
|  | Intentions |
|  | Goals |
|  | Reinforcement |
|  | Emotion |
